# Evolutionary analysis of proline-directed phosphorylation sites in the mammalian growth cone identified using phosphoproteomics

**DOI:** 10.1186/s13041-019-0476-x

**Published:** 2019-05-31

**Authors:** Michihiro Igarashi, Shujiro Okuda

**Affiliations:** 1 Department of Neurochemistry and Molecular Cell Biology, Niigata University Graduate School of Medical and Dental Sciences, 1-757 Asahimachi, Chuo-ku, Niigata, 951-8510 Japan; 2 Laboratory of Bioinformatics, Niigata University Graduate School of Medical and Dental Sciences, Niigata, Japan

**Keywords:** Growth cone, Phosphoproteomics, Bioinformatics, MAPK, Evolution, Vertebrates, *C. elegans*, *Drosophila*

## Abstract

The growth cone is essential for nerve growth and axon regeneration, which directly form and rearrange the neural network. Recently, to clarify the molecular signaling pathways in the growth cone that utilize protein phosphorylation, we performed a phosphoproteomics study of mammalian growth cone membranes derived from the developing rodent brain and identified > 30,000 phosphopeptides from ~ 1200 proteins. We found that the phosphorylation sites were highly proline directed and primarily mitogen-activated protein kinase (MAPK) dependent, due to particular activation of c-jun N-terminal protein kinase (JNK), a member of the MAPK family. Because the MAPK/JNK pathway is also involved in axon regeneration of invertebrate model organisms such *Caenorhabditis elegans* and *Drosophila*, we performed evolutionary bioinformatics analysis of the mammalian growth cone phosphorylation sites. Although these sites were generally conserved within vertebrates, they were not necessarily conserved in these invertebrate model organisms. In particular, high-frequency phosphorylation sites (> 20 times) were less conserved than low-frequency sites. Taken together, the mammalian growth cones contain a large number of vertebrate-specific phosphorylation sites and stronger dependence upon MAPK/JNK than *C. elegans* or *Drosophila*. We conclude that axon growth/regeneration likely involves many vertebrate-specific phosphorylation sites.

**Electronic supplementary material:**

The online version of this article (10.1186/s13041-019-0476-x) contains supplementary material, which is available to authorized users.

## Main text

The growth cone, a highly motile structure at the tip of extending axons in developing or regenerating neurons [1], is crucial for accurate synaptogenesis. Therefore, elucidating the molecular pathways for growth cone behavior is essential. At present, however, sufficient molecular information is not available regarding growth cones in the mammalian brain. We performed a proteomics analysis of mammalian growth cones and characterized approximately 1000 unique proteins [2]; the results revealed novel molecular mechanisms underlying nerve growth [1].

To further investigate molecular signaling in growth cones, we focused on protein phosphorylation, the most important regulatory mechanism in many cellular processes [3]. Phosphoproteomics is a powerful technique for comprehensive and quantitative identification of in vivo phosphorylation sites [3]. Thus, we performed phosphoproteomics analysis of the growth cone membrane (GCM). From among more than 30,000 phosphopeptides, this analysis identified ~ 4600 different phosphorylation sites from ~ 1200 proteins [4]. Surprisingly, proline (P)-directed phosphorylation [5] was predominant, with more than 60% of serine (S) or threonine (T) phosphorylation sites predicted to depend on P-directed kinases [4]. Bioinformatics analysis suggested that these frequent P-directed phosphorylation events were due to mitogen-activated protein kinase (MAPK) activation. In particular, we found that c-Jun N-terminal kinase (JNK) [6] was the major active member of the MAPK family and was responsible for several heavily phosphorylated sites [4].

The MAPK family includes extracellular signal–regulated kinase, p38, and JNK, among which JNK appeared to be the most likely kinase candidate for mammalian GCM phosphorylation. First, several recent reports showed that JNK is involved in multiple steps of mammalian brain development [7–11]. Second, JNK signaling is activated during axon regeneration, even in *Caenorhabditis elegans* [12]. Together, these observations suggest the importance of JNK signaling in a wide range of organisms.

**Fig. 1:**
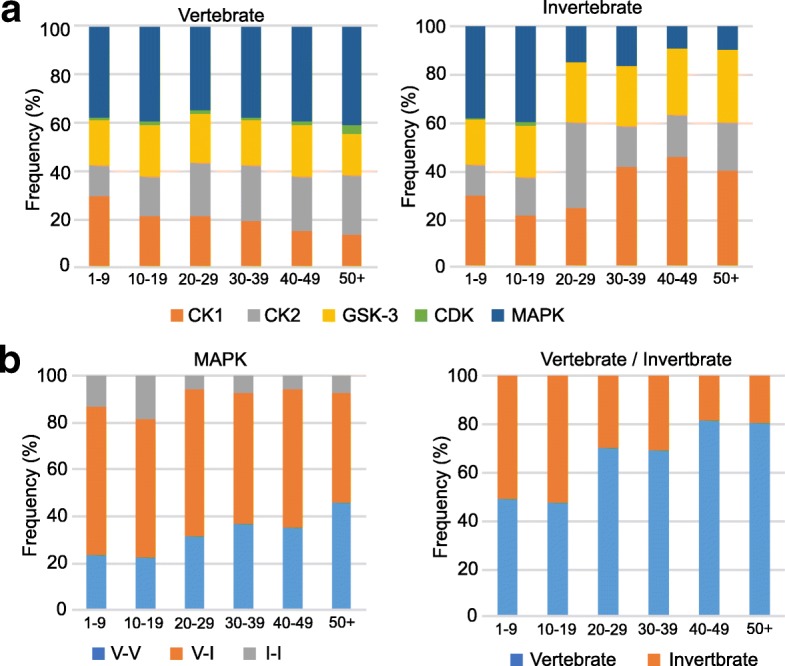
Evolutionary analysis of phosphosites in mammalian GCM using bioinformatics. **a** Distribution of kinases for P-directed phosphosites that were more conserved in vertebrates (*left*) than in invertebrates (*right*). Invertebrates: *C. elegans* and *Drosophila*; Vertebrates: lamprey, zebrafish, *Xenopus*, turtle, anole, chicken, and rat. The numbers on the bottom indicate the frequency of the identified phosphopeptide. CK1, CK2, GSK-3, CDK, and MAPK were predicted by KinasePhos 2.0 against phosphosites conserved in vertebrates to be higher than each phosphoproteomics score threshold. Note that the predicted MAPK-dependent sites were consistently evolutionarily conserved in vertebrates and accounted for more than 35% of all sites. In the high-score groups (≥20 phosphopeptides), the proportion of MAPK phosphorylation sites conserved in invertebrates was markedly lower. **b** Comparison of vertebrates and invertebrates regarding MAPK P-directed phosphosites (*left*) and MAPK P-directed phosphosite genes (*right*). *I-I*: the gene has emerged since invertebrates, and the protein has conserved SP/TP residues since invertebrates; *V-I*: the gene has emerged since invertebrates, but the protein has conserved SP/TP residues only in vertebrates; and *V-V*: the gene emerged first in vertebrates, and the protein has conserved SP/TP residues within vertebrates. The P-directed phosphosites with a high score that were phosphorylated by MAPK were conserved in vertebrates as both a phosphosite and also a gene. See the text. Note that as the phosphorylation scores increased in vertebrates, vertebrate-specific genes with highly MAPK-dependent sites (≥20 phosphopeptides) increased. The number with the detected frequency for each phosphopeptide is shown at the bottom (**a** and **b**)

Thus, to understand and characterize MAPK signaling in the GCM, we used bioinformatics to examine whether the phosphorylation sites of the mammalian GCM proteins that were identified using phosphoproteomics were conserved within a wide range of animals. If so, the signaling pathways involving those phosphosites were expected to be widely conserved from mammals to nematodes or insects. We first made an evolutionary comparison between vertebrates and invertebrates such as *C. elegans* and *Drosophila*, using comparative genomics data in “Ensembl” [13]. Surprisingly, we found that MAPK-dependent substrates with very frequently phosphorylated sites (detected ≥20 times) were conserved in vertebrates, but were less abundant in invertebrates; more than 70% of the very frequent sites were vertebrate specific (Fig. 1a; also see Additional file 1: Figure S1), suggesting the importance of JNK signaling in a wide range of animals. In addition, we classified these MAPK-dependent phosphoprotein-coding genes using KinasePhos 2.0 [14], a kinase prediction site, into three groups. We found that the vertebrate-specific phosphoproteins had more high-frequency sites compared to the evolutionarily older ones (Fig. 1b). Namely, highly MAPK-dependent sites were conserved within vertebrates, as were the genes encoding these sites, which newly emerged in vertebrates (Fig. 1b). Taken together, the data revealed that the substrates of MAPK signaling in rodent GCM included many vertebrate-specific proteins and vertebrate-specific phosphorylation sites, suggesting that axonal growth may be controlled by considerably distinct signaling pathways in vertebrates and invertebrates.

In the case of downstream genes of DLK-JNK signaling in *C. elegans* axon regeneration [15], few P-directed substrates were identified in our phosphoproteomics study [4], suggesting that different molecular mechanisms involving JNK play a role in mammalian axon growth/regeneration compared to *C. elegans,* although JNK is activated in neurons of both organisms. We conclude that the molecular signaling in mammalian growth cones for axon growth/regeneration may more frequently use evolutionarily newer phosphoproteins or phosphorylated sites that depend on MAPK/JNK, in addition to older ones that are also present in invertebrate phosphoproteins. These newly identified phosphorylated sites may have allowed more sophisticated signaling pathways that are more suitable for neural network formation in vertebrate brain, where the neuronal number is much larger than in invertebrates.

## Additional files

Additional file 1: **Figure S1**. Alignment of the P-directed GCM phosphoproteins emerging from the invertebrates. (PDF 9916 kb)

Additional file 2: Methods and the legend to Figure S1. (DOCX 16 kb)

## Data Availability

All data analyzed during this study, namely, the phosphopeptides identified using phosphoproteomics, are included in ref. [4] and its Additional file 2.

## Abbreviations

GCM: Growth cone membrane
JNK: c-jun *N*-terminal protein kinase
MAPK: Mitogen-activated protein kinase
