# Supplementary material for: Evolutionary analysis of proline-directed phosphorylation sites in the mammalian growth cone identified using phosphoproteomics
Source: Mol Brain. 2019 May 31;12:53. doi: 10.1186/s13041-019-0476-x (PMC6545026; doi:10.1186/s13041-019-0476-x)
Supplement: Supplementary file 1 — Figure S1. Alignment of the P-directed GCM phosphoproteins emerging from the invertebrates. (PDF 9916 kb) [file 13041_2019_476_MOESM1_ESM.pdf]

ENSRNOP00000023460[Map1b]@25

rattus_norvegicus	**Map1b**	PAATT**SP**SLS-H
gallus_gallus	MAP1B	PAA-P**SP**SLS-H
pelodiscus_sinensis	MAP1B	PAP-PCPSLS-H
anolis_carolinensis	MAP1B	AVP-P**SP**SLS-H
xenopus_tropicalis	MAP1B	PAA--**SP**SLS-H
xenopus_tropicalis	map1b	------------
danio_rerio	MAP1B	LRSTA**SP**TASTQ
drosophila_melanogaster	futsch	IVGPP**SP**LTG--
drosophila_melanogaster	dp	FVCTCKPDYTGD

ENSRNOP00000023460[Map1b]@27

rattus_norvegicus	**Map1b**	ATTSP**S**LS-HRF
gallus_gallus	MAP1B	A-PSP**S**LS-HRF
pelodiscus_sinensis	MAP1B	P-PCP**S**LS-HRF
anolis_carolinensis	MAP1B	P-PSP**S**LS-HRF
xenopus_tropicalis	MAP1B	A--SP**S**LS-HRF
xenopus_tropicalis	map1b	------------
danio_rerio	MAP1B	STASP**T**ASTQHF
drosophila_melanogaster	futsch	GPPSPLTG----
drosophila_melanogaster	dp	CTCKPDYTGDPF

ENSRNOP00000023460[Map1b]@336

rattus_norvegicus	**Map1b**	LEEER**S**QGSTS
gallus_gallus	MAP1B	LEEEQ**S**QGSTT
pelodiscus_sinensis	MAP1B	LEEEQ**S**QGSTT
anolis_carolinensis	MAP1B	LEEEQ**S**QGSTT
xenopus_tropicalis	MAP1B	LEEEQ**S**QGSTT
xenopus_tropicalis	map1b	VDEDP**S**EGPQA
danio_rerio	MAP1B	LEEEQ**S**QGSTA
drosophila_melanogaster	futsch	-----------
drosophila_melanogaster	dp	IDQHH**S**QCSCP

ENSRNOP00000023460[Map1b]@614

rattus_norvegicus	**Map1b**	SKEEQ**S**--**P**VKAE
gallus_gallus	MAP1B	TKEEQ---LGKSE
pelodiscus_sinensis	MAP1B	AKEEQ---LVKSE
anolis_carolinensis	MAP1B	AKEQQ---LAKSE
xenopus_tropicalis	MAP1B	IKQEQ--
xenopus_tropicalis	map1b	SKEEK--KP
danio_rerio	MAP1B	SKVEQ---THKTD
drosophila_melanogaster	futsch	TKEEEIQKHQRD--SQESEKKRKKSAEEEI-----EAAIAKVE
drosophila_melanogaster	dp	PHSEQ**T**TESTRDVPTTRPFEASTPSPASLETTVPSVTLETTTNVPIGSTGGQVTGQTTSSPSEVRTTIRVEESTLPSRSADRTTPSE

ENSRNOP00000023460[Map1b]@963

rattus_norvegicus	**Map1b**	SASKH**SP**TE----------------DE
gallus_gallus	MAP1B	DATKPYMEEARRIHDYS-------DE
pelodiscus_sinensis	MAP1B	GITKQDIDVTI---------------
anolis_carolinensis	MAP1B	GIRHQDMKDTIEIEKY---------E
xenopus_tropicalis	MAP1B	TAEEDGTEEDLDTDAY---------E
xenopus_tropicalis	map1b	--------------------------
danio_rerio	MAP1B	--------------------------
drosophila_melanogaster	futsch	SAVGSMKDESMSKEPSR-------RE
drosophila_melanogaster	dp	GSTGGQVTEQTTSSPSEVRTTIGLEE

ENSRNOP00000023460[Map1b]@985

rattus_norvegicus	**Map1b**	KEKRE**S**VASGD
gallus_gallus	MAP1B	TEKADY-ETSD
pelodiscus_sinensis	MAP1B	VEKAE**S**-EAGD
anolis_carolinensis	MAP1B	DEKAEFRDKGE
xenopus_tropicalis	MAP1B	EDIADI-EAAD
xenopus_tropicalis	map1b	-----------
danio_rerio	MAP1B	-------TLKD
drosophila_melanogaster	futsch	TQSKEAGSIKD
drosophila_melanogaster	dp	ESTRDVPTTRP

ENSRNOP00000023460[Map1b]@988

rattus_norvegicus	**Map1b**	`RESVASGDDR-------A`
gallus_gallus	MAP1B	`ADY-ETSDER-------A`
pelodiscus_sinensis	MAP1B	`AES-EAGDER-------A`
anolis_carolinensis	MAP1B	`AEFRDKGEEQ-------D`
xenopus_tropicalis	MAP1B	`ADI-EAADEQ-------A`
xenopus_tropicalis	map1b	`------------------`
danio_rerio	MAP1B	`----TLKDEQ-------A`
drosophila_melanogaster	futsch	`KEAGSIKDEKS----PLA`
drosophila_melanogaster	dp	`RDVPTTRPFEASTPSP-A`

ENSRNOP00000023460[Map1b]@1009

rattus_norvegicus	**Map1b**	GEAEQ**S**------------EEEGE
gallus_gallus	MAP1B	AETEE**T**------------EE---
pelodiscus_sinensis	MAP1B	AETEE**T**------------EEE-A
anolis_carolinensis	MAP1B	AETEE**T**------------EEE-A
xenopus_tropicalis	MAP1B	GEADE**T**------------EEE--
xenopus_tropicalis	map1b	-----------------------
danio_rerio	MAP1B	G-TDEP------------EYL--
drosophila_melanogaster	futsch	AESVKD------------EAEKS
drosophila_melanogaster	dp	SETTTNVPIGSTGGQVTGQTTAP

ENSRNOP00000023460[Map1b]@1148

rattus_norvegicus	**Map1b**	NEETE**S**-----------**P**SQEF
gallus_gallus	MAP1B	NEESE**S**-----------**P**SQEY
pelodiscus_sinensis	MAP1B	NEESE**S**-----------**P**SQEF
anolis_carolinensis	MAP1B	NEESE**S**-----------**P**SQEY
xenopus_tropicalis	MAP1B	NEESE**S**-----------**P**SQEY
xenopus_tropicalis	map1b	--EPE**S**-----------**P**N---
danio_rerio	MAP1B	NDESD**S**-----------**P**SQDF
drosophila_melanogaster	futsch	KSKEE**S**RRESVAEKSPLPSKEA
drosophila_melanogaster	dp	SSPSEVRTTIRVEESTLPSRST

ENSRNOP00000023460[Map1b]@1201

rattus_norvegicus	**Map1b**	SA---STI**SP**PSSM
gallus_gallus	MAP1B	SA---STI**SP**PSSL
pelodiscus_sinensis	MAP1B	SA---STL**SP**PSSL
anolis_carolinensis	MAP1B	SA---STI**SP**PSSL
xenopus_tropicalis	MAP1B	SA---STL**SP**PSSL
xenopus_tropicalis	map1b	--
danio_rerio	MAP1B	SA---STI**SP**PSSL
drosophila_melanogaster	futsch	KGD--------------------QSP--LKEV**S**RPESV
drosophila_melanogaster	dp	SYFRNHYKCSNRFNRSADRTTPSESPETPTLPSDFTTRPHSEQTTESTRDVPTTRPFEASTPSPASLETTVPSVTSETTTNVPIGSTGGQVTGQTTAPPSEV

ENSRNOP00000023460[Map1b]@1257

rattus_norvegicus	**Map1b**	SPSPP**S**---**P**I-EKT
gallus_gallus	MAP1B	SLSPP**S**---**P**V-AKT
pelodiscus_sinensis	MAP1B	SPSLP**S**---**P**V-AKT
anolis_carolinensis	MAP1B	SPGLL**S**---**P**A-AKT
xenopus_tropicalis	MAP1B	SPVPP**S**---**P**I-SKT
xenopus_tropicalis	map1b	SLAPI**S**---**P**L-AKT
danio_rerio	MAP1B	---PF-VRS
drosophila_melanogaster	futsch	SIKAS**S**---**P**RDEKS
drosophila_melanogaster	dp	TPSPA**S**LETTVPSVTSETTTNVPIGSTGGQVTEQTTSSPSEVRTTIGLEESTLPSRSTDRTSPSESPETPTTLPSDFITRPHSDQTTESTRDVPTTRPFEAST

ENSRNOP00000023460[Map1b]@1305

rattus_norvegicus	**Map1b**	EEHCA**SP**---------EEKT
gallus_gallus	MAP1B	EEHCA**SP**---------EDKT
pelodiscus_sinensis	MAP1B	EEHCV**SP**---------DDKT
anolis_carolinensis	MAP1B	EEQCA**SP**---------DDKT
xenopus_tropicalis	MAP1B	NEQCA**SP**---------EEKT
xenopus_tropicalis	map1b	---YG**S**S---------EERT
danio_rerio	MAP1B	----Y**SP**---------DEKT
drosophila_melanogaster	futsch	AETVS**SP**--------IEEAT
drosophila_melanogaster	dp	EQTTS**SP**SEVRTTIRVEEST

ENSRNOP00000023460[Map1b]@1315

rattus_norvegicus	**Map1b**	TLE--VV**SP**SQSV
gallus_gallus	MAP1B	TLE--VA**SP**SQSA
pelodiscus_sinensis	MAP1B	TLE--VV**SP**SQSA
anolis_carolinensis	MAP1B	TLE--VV**SP**SQSA
xenopus_tropicalis	MAP1B	TLE--VT**SP**SQSA
xenopus_tropicalis	map1b	TLE--MA**SP**A---
danio_rerio	MAP1B	TLE---GPL**SP**Q---
drosophila_melanogaster	futsch	TMEFSKI---------------------------EVVEKSSLALSLQ-----------------------------GGSGGKL--QTDSSP------VDVAEGDF-----SHAVASVSTVTPT-----------------------LTKPAELA----------------------QIGAA------KTVSSPLDEALRT------PSAPEHISRAD**SP**AECA
drosophila_melanogaster	dp	TLP-SRSTDRTTPSESPETPTTLPSDFTTRPHSDQTTESSRDVPTTQPFESSTPRPVTLETAVPPVTSETTTNVPIGSTGGQVTEQTTPSPSEVRTTIRIEESTFPSRSTDRTTPSESPETPTTLPSDFTTRPHSDQTTESTRDVPTTRPFESSTPRPVTLEIAVPPVTSETTTNVAIGSTGGQVTEQTTSSP--SEVRTTIRVEESTLPSRSTDRT**TP**SESP

ENSRNOP00000023460[Map1b]@1382

rattus_norvegicus	**Map1b**	VPD--SE**SP**--IEKV
gallus_gallus	MAP1B	VPD--SE**SP**--IEKV
pelodiscus_sinensis	MAP1B	VPD--SE**SP**--IEKV
anolis_carolinensis	MAP1B	VPD--SE**SP**--FEKV
xenopus_tropicalis	MAP1B	VPD--SE**SP**--IEKV
xenopus_tropicalis	map1b	LPS--GD**S**R---GLN
danio_rerio	MAP1B	VPV--**S**QP--GKK
drosophila_melanogaster	futsch	CPA---EERPE**SP**-------------------AESAKDAAE---SVEKS
drosophila_melanogaster	dp	VPSVTLETTTNVPIGSTGGQVTGQTTATPSEVRTTIGVEESTLPSRSTDRTTPSE**SP**ETPTTLPSDFTTRPHSDQTTESTRDVPTTRPFEASTPRPVTLETAVPSVTSETTTNVPIGSTVTSETTTNVPIGSTGGQVAGQTTAPPSEVRTTIRVEES

ENSRNOP00000023460[Map1b]@1393

rattus_norvegicus	**Map1b**	LSPLR**S**--**P**PLIG
gallus_gallus	MAP1B	LSPLR**S**--**P**PLIG
pelodiscus_sinensis	MAP1B	LSPLR**S**--**P**PLIG
anolis_carolinensis	MAP1B	LSPLR**S**--**P**PLIG
xenopus_tropicalis	MAP1B	LSPLR**S**--**P**PLIG
xenopus_tropicalis	map1b	MSSWKV--PP---
danio_rerio	MAP1B	QSPI---
drosophila_melanogaster	futsch	KDASRP--PSVVE
drosophila_melanogaster	dp	TLPSR**S**ADRTTPSESPETPTTLPSDFTTRPHSEQTTESTRDVPTTRPFEASTPSPASLETTVPSVTS

ENSRNOP00000023460[Map1b]@1420

rattus_norvegicus	**Map1b**	GRRSE**SP**FEG--K
gallus_gallus	MAP1B	TRDYE**SP**SEG--K
pelodiscus_sinensis	MAP1B	DRQSE**S**HFER--K
anolis_carolinensis	MAP1B	HRETP**SP**FE---K
xenopus_tropicalis	MAP1B	ARHD---FEKVTD
xenopus_tropicalis	map1b	------------K
danio_rerio	MAP1B	-------FEFD--
drosophila_melanogaster	futsch	SRPA-**S**VVESV-K
drosophila_melanogaster	dp	APPSVRTTETIVK

ENSRNOP00000023460[Map1b]@1432

rattus_norvegicus	**Map1b**	GKQGF**S**-DKESP
gallus_gallus	MAP1B	EKEK-**SP**VQMSP
pelodiscus_sinensis	MAP1B	EKEE-**SP**VQISP
anolis_carolinensis	MAP1B	EKDG-**SP**LQKSP
xenopus_tropicalis	MAP1B	DNEK-**SP**TRISP
xenopus_tropicalis	map1b	SQEN-**S**NSSQGR
danio_rerio	MAP1B	------------
drosophila_melanogaster	futsch	EHDK-AESRRES
drosophila_melanogaster	dp	THPAV**SP**-DTTI

ENSRNOP00000023460[Map1b]@1436

rattus_norvegicus	**Map1b**	FSDKE**SP**VSDL
gallus_gallus	MAP1B	SPVQM**SP**ASEA
pelodiscus_sinensis	MAP1B	SPVQI**SP**VSEI
anolis_carolinensis	MAP1B	SPLQK**SP**ASEV
xenopus_tropicalis	MAP1B	SPTRI**SP**VSEM
xenopus_tropicalis	map1b	SNSSQGRQTSC
danio_rerio	MAP1B	--------SKL
drosophila_melanogaster	futsch	AESRRESIAKV
drosophila_melanogaster	dp	VSPDT**T**IPSEI

ENSRNOP00000023460[Map1b]@1465

rattus_norvegicus	**Map1b**	IKEDF**SP**EKKA
gallus_gallus	MAP1B	IKESF**S**LERKM
pelodiscus_sinensis	MAP1B	TATGFGLEKNM
anolis_carolinensis	MAP1B	SKSDFVLEKKK
xenopus_tropicalis	MAP1B	SKE-FYSDAAT
xenopus_tropicalis	map1b	-----------
danio_rerio	MAP1B	-------DKKI
drosophila_melanogaster	futsch	SRRESVVESSK
drosophila_melanogaster	dp	TPRGQVTERTT

ENSRNOP00000023460[Map1b]@1494

rattus_norvegicus	**Map1b**	LGGDG**SP**TQVD
gallus_gallus	MAP1B	LTGDL**SP**TQID
pelodiscus_sinensis	MAP1B	LAGDV**SP**TQID
anolis_carolinensis	MAP1B	L-GEI**SP**TQVD
xenopus_tropicalis	MAP1B	YAGDF**SP**TQIS
xenopus_tropicalis	map1b	----L**SP**----
danio_rerio	MAP1B	I-ESL**S**ETK--
drosophila_melanogaster	futsch	PVPRE**S**KSPLD
drosophila_melanogaster	dp	PVSDNLPTTIS

ENSRNOP00000023460[Map1b]@1613

rattus_norvegicus	**Map1b**	RPMSI--SPPDFS
gallus_gallus	MAP1B	RPMSI--SPPDFS
pelodiscus_sinensis	MAP1B	RPMSI--SPPDFS
anolis_carolinensis	MAP1B	RPMSI--SPPDFS
xenopus_tropicalis	MAP1B	RPMSI--SPPDFS
xenopus_tropicalis	map1b	R--NV--SPVAHA
danio_rerio	MAP1B	RPMSI--SP-DVS
drosophila_melanogaster	futsch	RPESVL--GSLKDEG
drosophila_melanogaster	dp	RPETVCEPNPCGPNSICRSVEGHPTCSCQVGYFGAPPQCRPECVVSSECAQHLSCINQKCMDPCVGTCGFNAKCQVNNHNPICSCPANYEGNPFEQCMPKPAEPTRNVDPCLPSPCGSNSICRNVNNRAECSCAPGMFGAPPNCRPECVINQDCPSNRACIRQRCEDPCIGICGFNAVCSTQNHQPKCSCIESFEGDPYTACKMREIVVLDPPTDPCYPSPCGANAICRVRNGAGSCSCIQNYFGDPYINCRPECVQNSDCPNNRACINMKCRDPCANACGFNAICRVAHHQPVCSCEPHLTGNPLRACVERPSNMYLPLPKDPCRPSPCGLFSTCHVVGERPVCACLPDYMGAPPNCK

ENSRNOP00000023460[Map1b]@1646

rattus_norvegicus	**Map1b**	EFG---QE**SP**EHSL
gallus_gallus	MAP1B	EFG---QE**SP**EQSL
pelodiscus_sinensis	MAP1B	EFS---QE**SP**EQSL
anolis_carolinensis	MAP1B	EFG---QD**SP**EHSL
xenopus_tropicalis	MAP1B	EFS---QE**SP**EQSF
xenopus_tropicalis	map1b	---PTHSV
danio_rerio	MAP1B	E---QE**SP**EHSF
drosophila_melanogaster	futsch	KDGK--SPVASKEASRPASVAENA---------------KDSADESKEQRPESL--PQSKAGSIKDEK----------SPLASKDEAEKS---KEE**S**RRESV
drosophila_melanogaster	dp	CPGNLACINLRCRDPCVGTCGIQTTCLVNNHRPICRCIDGYAGDPFSECSPKINVPVQVAQPCNPSPCGANAVCKERNGVGSCSCLPEYNGDPYTECRPECVLNSDCSKNRACLNNKCRDPCPGVCGVSAECHVINHAPSCSCPSGFTGNPSQFCREIPRLPAPVEPCRPSPCGPYSQCREVNGHAVCSCVTNYIGTPPACRPECSVSSECAQDRACVNQRCADPCPGTCGNEAICKVTNHNPICSCPAGYSGDPFVRCAPWQEEPEQPK

ENSRNOP00000023460[Map1b]@1659

rattus_norvegicus	**Map1b**	DF---SRQ**SP**DHPT
gallus_gallus	MAP1B	DF---SRQ**SP**EYPT
pelodiscus_sinensis	MAP1B	DF---SRQ**SP**EHPT
anolis_carolinensis	MAP1B	DF---SRQ**SP**EHPI
xenopus_tropicalis	MAP1B	DF---SKQ**SP**EHPA
xenopus_tropicalis	map1b	D---
danio_rerio	MAP1B	DF---SKQ**SP**DHPS
drosophila_melanogaster	futsch	QFPLVSKEVSR------------------------------------PASVA---ESVKDE--AEKSKEES---PLMSKEASRPAS
drosophila_melanogaster	dp	ENPCVPSPCGRNSQCRVVGETGVCSCLPNFVGRAPNCRPECTINTECPANLACINERCQDPCPGSCGFNAFCSVVNHSPICTCDSGYTGDPFAGCNPQPPAIPDERLTPCQPSPCGPNAECRERNGAGSCTCLPEYFGDPYSGCRPECVVNSDCSRDKSCVNQKCVDPCPGVCGLNAQCRVSNHLPSCSCLAGYTGNPSSACREIPQLPPPPERDENPCRPS

ENSRNOP00000023460[Map1b]@1683

rattus_norvegicus	**Map1b**	TEVDY**SP**SDIQ
gallus_gallus	MAP1B	TEVDY**SP**SDIQ
pelodiscus_sinensis	MAP1B	TEVDF**SP**SDTQ
anolis_carolinensis	MAP1B	TEVDF**SP**SDMQ
xenopus_tropicalis	MAP1B	TEVEY**SP**SDPH
xenopus_tropicalis	map1b	SERSA**SP**HD--
danio_rerio	MAP1B	TEVDD**SP**LD-E
drosophila_melanogaster	futsch	TSVAE**S**VKDEA
drosophila_melanogaster	dp	TESPS**SP**CEPS

ENSRNOP00000023460[Map1b]@1765

rattus_norvegicus	**Map1b**	S-------EKVQ**S**LE--GEK
gallus_gallus	MAP1B	S-------EKVQ**S**L---GEK
pelodiscus_sinensis	MAP1B	S-------EKMQ**S**L---GEK
anolis_carolinensis	MAP1B	S-------EKMQ**S**L---GEK
xenopus_tropicalis	MAP1B	L-------EHMYFME--G-K
xenopus_tropicalis	map1b	---
danio_rerio	MAP1B	--K
drosophila_melanogaster	futsch	D-------EAEK**S**KEES-------------------RRESVAEKSP---------------------------LASKEASRPASVAES--VKDEAEKSKEESRRESVAEKSPL------PSKE---------------------ASRPTSVAESVKDEADKSK---EESRRESGAE
drosophila_melanogaster	dp	DPCPGVCGQNSQCDVSNHIPICSCLQGYTGDPFVHCRHETPVAKDPCQPNPCGPNSLCHISGQGPVCACQPGMLGSPPACKPECIVSSECSLHTACVNRKCVDPCPGACGQFARCQVINHNPSCSCNTGYTGDPFTRCYQEERKPPTTPDNPCQPSPCGPNSECKVLNGNAACSCAATFIGTPPSCRPEC---SINPECPPTKACIRQKCSDPCVNACGFNARCNVANHQPICTCDVGYTGDPFTGCQKEQERIVNEQ

ENSRNOP00000023460[Map1b]@1772

rattus_norvegicus	**Map1b**	E--GEKL**SP**---KSDI
gallus_gallus	MAP1B	---GEKL**SP**---KSDL
pelodiscus_sinensis	MAP1B	---GEKL**SP**---KSDL
anolis_carolinensis	MAP1B	---GEKL**SP**---KSDL
xenopus_tropicalis	MAP1B	E--G-KL**SP**---ETDA
xenopus_tropicalis	map1b	--
danio_rerio	MAP1B	---KQ**SP**--SKQ
drosophila_melanogaster	futsch	EES-------------------RRESVAEKSP---------------------------LASKEASRPASVAES--VKDEAEKSKEESRRESVAEKSPL------PSKE---------------------ASRPTSVAESVKDEADKSK---EESRRESGAEK**SP**LA----------------------------SMEASRPTSVAES------------VKDE---TEKSK---EESRRESVTEK
drosophila_melanogaster	dp	VSNHIPICSCLQGYTGDPFVHCRHETPVAKDPCQPNPCGPNSLCHISGQGPVCACQPGMLGSPPACKPECIVSSECSLHTACVNRKCVDPCPGACGQFARCQVINHNPSCSCNTGYTGDPFTRCYQEERKPPTTPDNPCQPSPCGPNSECKVLNGNAACSCAATFIGTPPSCRPEC---SINPECPPTKACIRQKCSDPCVNACGFNARCNVANHQPICTCDVGYTGDPFTGCQKEQERIVNEQV**TP**CEPNPCGSNAVCRERNGIGSCQCLPDHFGDPYQSCRPECVRHSDCASNKACQQQKCRDPCPGTCGSNADCSVTNHLPTCTCRIGYTGDPYRYCHVEPPQLPARVTEPSQPCRPSPCGPNSQCRELNGQAVCSCLELYIGLPPNCRPECVLSTECPTDKACISQRCQDPCPGTCGINAECR---VRNH

ENSRNOP00000023460[Map1b]@1778

rattus_norvegicus	**Map1b**	P---KSDI**SP**L---TPR
gallus_gallus	MAP1B	P---KSDL**SP**L---TPR
pelodiscus_sinensis	MAP1B	P---KSDL**SP**L---TTR
anolis_carolinensis	MAP1B	P---KSDL**SP**L---TPR
xenopus_tropicalis	MAP1B	P---ETDA**SP**L---TPT
xenopus_tropicalis	map1b	---
danio_rerio	MAP1B	P--SKQ**SP**L---SLD
drosophila_melanogaster	futsch	PLA----------------------------SMEASRPTSVAES-----------VKDE--TEKSK---EESRRESVTEK**SP**LP---SKEASRPTSVA---ESVKDEAEKSKEESRR
drosophila_melanogaster	dp	PCEPNPCGSNAVCRERNGIGSCQCLPDHFGDPYQSCRPECVRHSDCASNKACQQQKCRDPCPGTCGSNADCSVTNHLPTCTCRIGYTGDPYRYCHVEPPQLPARVTEPSQPCRPSPCGPNSQCRELNGQAVCSCLELYIGLPPNCRPECVLSTECPTDKACISQRCQDPCPGTCGINAECR---VRNH**SP**LCQCRQGFTGDSFTRCYPLPPPPPVIERVERDPCLPSPCGLNSQCRNVQGVPSCTCLPDFLGAPPNCRPECTISAECPSNLACIRERCIDPCPGSCGYAAE

ENSRNOP00000023460[Map1b]@1786

rattus_norvegicus	**Map1b**	TPRES**SP**TYSP
gallus_gallus	MAP1B	TPRES**SP**LYSP
pelodiscus_sinensis	MAP1B	TTRES**SP**LFSP
anolis_carolinensis	MAP1B	TPRES**SP**LYSP
xenopus_tropicalis	MAP1B	TPTPS**S**LKQSP
xenopus_tropicalis	map1b	-----------
danio_rerio	MAP1B	SLDSSLVLGRP
drosophila_melanogaster	futsch	SRRESVAEKSP
drosophila_melanogaster	dp	AAECSVVNHTP

ENSRNOP00000023460[Map1b]@1874

rattus_norvegicus	**Map1b**	`ESTTES--------------------------------------PDEED`
gallus_gallus	MAP1B	`EKTTRS--------------------------------------PEDIS`
pelodiscus_sinensis	MAP1B	`GKTTKS--------------------------------------PEALS`
anolis_carolinensis	MAP1B	`GKITRS--------------------------------------PEDIG`
xenopus_tropicalis	MAP1B	`----QS--------------------------------------P----`
xenopus_tropicalis	map1b	`-------------------------------------------------`
danio_rerio	MAP1B	`----MS--------------------------------------P----`
drosophila_melanogaster	futsch	`ESEKSS--------------------------------------PDQKS`
drosophila_melanogaster	dp	`ECVTSSECPTNQACIQQKCRDPCPGLCGQSAECRVLSHTPSCVCPEGME`

ENSRNOP00000023460[Map1b]@1908

rattus_norvegicus	**Map1b**	ERTIK**SP**CDSG
gallus_gallus	MAP1B	GKITR**SP**EATD
pelodiscus_sinensis	MAP1B	EKTTK**S**LEAMD
anolis_carolinensis	MAP1B	GKTTR**SP**ETDS
xenopus_tropicalis	MAP1B	-----------
xenopus_tropicalis	map1b	-----------
danio_rerio	MAP1B	-----------
drosophila_melanogaster	futsch	PSPSQ**S**AASHE
drosophila_melanogaster	dp	QDPCPGTCGQN

ENSRNOP00000023460[Map1b]@1925

rattus_norvegicus	**Map1b**	EKTTK**TP**-EDGG
gallus_gallus	MAP1B	GKMTR**SP**-GDTD
pelodiscus_sinensis	MAP1B	GKTTR**SP**-ETVS
anolis_carolinensis	MAP1B	GKAIK**SP**-EKFD
xenopus_tropicalis	MAP1B	--ILH**S**T-NPFV
xenopus_tropicalis	map1b	------------
danio_rerio	MAP1B	DKFST**S**S-SSYS
drosophila_melanogaster	futsch	HKAEK**S**S-RPES
drosophila_melanogaster	dp	NPCQP**SP**CGPNS

ENSRNOP00000023460[Map1b]@1942

rattus_norvegicus	**Map1b**	EKTTR**T**--**P**EEGG
gallus_gallus	MAP1B	QKTTV**S**--**P**GDTD
pelodiscus_sinensis	MAP1B	EKTTM**S**--SEAVE
anolis_carolinensis	MAP1B	EKSLGL--SESLI
xenopus_tropicalis	MAP1B	ERE--
xenopus_tropicalis	map1b	---
danio_rerio	MAP1B	EST--
drosophila_melanogaster	futsch	EKDM---
drosophila_melanogaster	dp	EQAIC**S**CLPEYVGAPPVCRPECTISSECPADKACVNQKCVDPCPNTCGDQAICRVVNHSPICSCRAG

ENSRNOP00000023460[Map1b]@2027

rattus_norvegicus	**Map1b**	TK--TTR**SP**DTS-A
gallus_gallus	MAP1B	GR--TTR**SP**E-ATD
pelodiscus_sinensis	MAP1B	EK--STS**SP**EAAVG
anolis_carolinensis	MAP1B	GK--TTR**SP**DKA--
xenopus_tropicalis	MAP1B	--
xenopus_tropicalis	map1b	--
danio_rerio	MAP1B	DG--GSIGPETS--
drosophila_melanogaster	futsch	DSSR--------RESLSSLLAEKGGIAT-----------------------------NTSLKEDTS-A
drosophila_melanogaster	dp	QKCNDPCPGSCGRNAQCSVVNHNPFCTCLPRFTGNPFVGCQQIIEPPRQDIVPQDCRPSPCGPNSECR

ENSRNOP00000015386[Ank2]@1467

rattus_norvegicus	**Ank2**	VPVLA**SP**DLLS
pelodiscus_sinensis	ANK2	-----------
anolis_carolinensis	ANK2	VPVLA**SP**DLLS
xenopus_tropicalis	ank2	VPVLA**SP**DLLS
drosophila_melanogaster	Ank2	VHVVE**S**SSIEE
caenorhabditis_elegans	F22G12.4	-----------
caenorhabditis_elegans	unc-44	SPVPEEPEMEE

ENSRNOP00000015386[Ank2]@1816

rattus_norvegicus	**Ank2**	K------------NEKH**SP**VSPS
pelodiscus_sinensis	ANK2	-----------------------
anolis_carolinensis	ANK2	--------------SKL**S**--SPS
xenopus_tropicalis	ank2	--------------------TPV
drosophila_melanogaster	Ank2	IDEEVLAKPGGSSKSVVETDKPS
caenorhabditis_elegans	F22G12.4	-----------------------
caenorhabditis_elegans	unc-44	-------------SSHVESERPI

ENSRNOP00000015386[Ank2]@1828

rattus_norvegicus	**Ank2**	KA------------------------ERH**SP**VFS-----------G
pelodiscus_sinensis	ANK2	--
anolis_carolinensis	ANK2	KK------------------------PAV**SP**TS-------------
xenopus_tropicalis	ank2	DD------------------------EEI**S**HVI-------------
drosophila_melanogaster	Ank2	KEYSDDETEDEIDFPKPQDKPFKEATPSV**TP**VSTIPDVKAIDFVSF
caenorhabditis_elegans	F22G12.4	--
caenorhabditis_elegans	unc-44	ES------------------------PAV**S**----------------

ENSRNOP00000015386[Ank2]@2054

rattus_norvegicus	**Ank2**	TPVIK**TP**EAVP
pelodiscus_sinensis	ANK2	-----------
anolis_carolinensis	ANK2	QQSSK**T**TGKKS
xenopus_tropicalis	ank2	TSEHRVCELSK
drosophila_melanogaster	Ank2	SPLHPEEKLKS
caenorhabditis_elegans	F22G12.4	-----------
caenorhabditis_elegans	unc-44	TEEFDHSHPES

ENSRNOP00000015386[Ank2]@2096

rattus_norvegicus	**Ank2**	MDLQ--I**SP**DRKT
pelodiscus_sinensis	ANK2	---
anolis_carolinensis	ANK2	PDLQ--I**SP**DRKT
xenopus_tropicalis	ank2	VDPQ--I**SP**DRKT
drosophila_melanogaster	Ank2	DEIEIPKPLDKPISHPTSLVTGVTFGGDKSPLHPEEKPKSPEKKDEKVLAKPDGSSKSVVETDKP**SP**KEYS
caenorhabditis_elegans	F22G12.4	---
caenorhabditis_elegans	unc-44	DEPED-----------------------------EQEHQGPH---------------------SPAPSSHT

ENSRNOP00000015386[Ank2]@2238

rattus_norvegicus	**Ank2**	TIKVE**TP**---TDIH
pelodiscus_sinensis	ANK2	--
anolis_carolinensis	ANK2	ASAVQRK---AETH
xenopus_tropicalis	ank2	---LK**TP**---SNIV
drosophila_melanogaster	Ank2	PKPLDKPISHPTSLVTGVTFGGDGSPLHPEEKPKSPEKKDEKVLAKPDGSSKSVVETDKP
caenorhabditis_elegans	F22G12.4	--
caenorhabditis_elegans	unc-44	PSVIE**T**K---TTTT

ENSRNOP00000015386[Ank2]@2369

rattus_norvegicus	**Ank2**	QG--VIR**SP**---QGLE
pelodiscus_sinensis	ANK2	--
anolis_carolinensis	ANK2	EGA-SLEHHAI--------------------------------------SKGLE
xenopus_tropicalis	ank2	EHSVSVKGH---QGLE
drosophila_melanogaster	Ank2	DDE-------IDFPKPLDKPISHPTSLVTGVTFGVDKSPLHPEEKPKLPEKKDE
caenorhabditis_elegans	F22G12.4	--
caenorhabditis_elegans	unc-44	DGE-GLG**S**KVLGFAK--------KAGMVAG---GVVAAPV---ALAAVGAKAAY

ENSRNOP00000015386[Ank2]@2547

rattus_norvegicus	**Ank2**	EKKRF**TP**EEEM
pelodiscus_sinensis	ANK2	-----------
anolis_carolinensis	ANK2	PKKRF**TP**EEEM
xenopus_tropicalis	ank2	PKRRF**TP**EEEM
drosophila_melanogaster	Ank2	DEKVLPKPDDS
caenorhabditis_elegans	F22G12.4	-----------
caenorhabditis_elegans	unc-44	KSVKH**T**TETTT

ENSRNOP00000015386[Ank2]@3349

rattus_norvegicus	**Ank2**	D--GRSKSESDA-S
pelodiscus_sinensis	ANK2	--------------
anolis_carolinensis	ANK2	----RSKSESDTSV
xenopus_tropicalis	ank2	----RSKSESDSCS
drosophila_melanogaster	Ank2	DDSSKSVVETDKPS
caenorhabditis_elegans	F22G12.4	--------------
caenorhabditis_elegans	unc-44	----DPEVEKDVVE

ENSRNOP00000015386[Ank2]@3368

rattus_norvegicus	**Ank2**	PVKAR**S**YIETE
pelodiscus_sinensis	ANK2	-----------
anolis_carolinensis	ANK2	SVKAR**S**YVEAE
xenopus_tropicalis	ank2	SVKTK**S**CTETF
drosophila_melanogaster	Ank2	DDSSK**S**VVETD
caenorhabditis_elegans	F22G12.4	-----------
caenorhabditis_elegans	unc-44	YYKSE**SP**VQTE

ENSRNOP00000015386[Ank2]@3698

rattus_norvegicus	**Ank2**	PAAAL**SP**QMHQ
pelodiscus_sinensis	ANK2	SVAELLRQAHK
anolis_carolinensis	ANK2	SVVELLRQTHK
xenopus_tropicalis	ank2	SVVEMLRQLQK
drosophila_melanogaster	Ank2	NLAALAEQSDS
caenorhabditis_elegans	F22G12.4	-----------
caenorhabditis_elegans	unc-44	EAAAQKEQDEK

ENSRNOP00000015386[Ank2]@3751

rattus_norvegicus	**Ank2**	PEDIS**TP**PEEA
pelodiscus_sinensis	ANK2	----------SA
anolis_carolinensis	ANK2	STAVS------
xenopus_tropicalis	ank2	RTQVSQSPQET
drosophila_melanogaster	Ank2	MKEVSVDSDEA
caenorhabditis_elegans	F22G12.4	-----------
caenorhabditis_elegans	unc-44	TTATSQAPSRL

ENSRNOP00000048442[Ncam1]@769

rattus_norvegicus	**Ncam1**	GKAAF**S**KDES-------K
gallus_gallus	NCAM1	GKAAF**S**KDES-------K
pelodiscus_sinensis	NCAM1	GKAAF**S**KDES-------K
anolis_carolinensis	NCAM1	GKAAF**S**KDES-------K
xenopus_tropicalis	ncam1	GKAAF**S**KDES-------K
drosophila_melanogaster	CG8964	------------------
drosophila_melanogaster	Fas2	AKLGSGQ--------LVK
drosophila_melanogaster	CG33543	------------------
drosophila_melanogaster	zormin	DRYKY**S**GSESDVEPGIRK
drosophila_melanogaster	CG15630	------------------
drosophila_melanogaster	otk	RRSKT**S**MDDIE-------
caenorhabditis_elegans	ncam-1	GR----------------

ENSRNOP00000048442[Ncam1]@773

rattus_norvegicus	**Ncam1**	FSKDE**S**-------KEPIV
gallus_gallus	NCAM1	FSKDE**S**-------KEPIV
pelodiscus_sinensis	NCAM1	FSKDE**S**-------KEPIV
anolis_carolinensis	NCAM1	FSKDE**S**-------KEPIV
xenopus_tropicalis	ncam1	FSKDE**S**-------KEPIV
drosophila_melanogaster	CG8964	------------------
drosophila_melanogaster	Fas2	SGQ--------LVKEP--
drosophila_melanogaster	CG33543	------------------
drosophila_melanogaster	zormin	YSGSE**S**DVEPGIRKQPQL
drosophila_melanogaster	CG15630	------------------
drosophila_melanogaster	otk	TSMDDIE-----------
caenorhabditis_elegans	ncam-1	------------------

ENSRNOP00000048442[Ncam1]@787

rattus_norvegicus	**Ncam1**	TEEER**TP**NHDG
gallus_gallus	NCAM1	TEEER**TP**NHDG
pelodiscus_sinensis	NCAM1	TEEER**TP**NHDG
anolis_carolinensis	NCAM1	TEEER**TP**NHDG
xenopus_tropicalis	ncam1	TEEER**TP**NHDG
drosophila_melanogaster	CG8964	-QEHDHHENE-
drosophila_melanogaster	Fas2	-----------
drosophila_melanogaster	CG33543	-----------
drosophila_melanogaster	zormin	TEEHS**S**YRKSE
drosophila_melanogaster	CG15630	-----------
drosophila_melanogaster	otk	EEEQDQHNQS-
caenorhabditis_elegans	ncam-1	-----------

ENSRNOP00000048442[Ncam1]@802

rattus_norvegicus	**Ncam1**	EPNET**T**------------**P**LTEP
gallus_gallus	NCAM1	EPNET**T**------------**P**LTEP
pelodiscus_sinensis	NCAM1	EPNET**T**------------**P**LTEP
anolis_carolinensis	NCAM1	EPNET**T**------------**P**LTEP
xenopus_tropicalis	ncam1	EPNET**T**------------**P**LTEP
drosophila_melanogaster	CG8964	-----------------------
drosophila_melanogaster	Fas2	-----------------------
drosophila_melanogaster	CG33543	-----------------------
drosophila_melanogaster	zormin	ETKSS**S**TMGGAPQLQTQFPKLQP
drosophila_melanogaster	CG15630	-----------------------
drosophila_melanogaster	otk	---------GLEQL---------
caenorhabditis_elegans	ncam-1	-----------------------

ENSRNOP00000052108[Epb4.1l1]@30

rattus_norvegicus	**Epb4.1l1**	AAAVT**TP**VTPA
drosophila_melanogaster	cora	-----------
drosophila_melanogaster	CG5022	-----------

ENSRNOP00000052108[Epb4.1l1]@33

rattus_norvegicus	**Epb4.1l1**	VTTPV**TP**AGHS
drosophila_melanogaster	cora	-----------
drosophila_melanogaster	CG5022	-----------

ENSRNOP00000052108[Epb4.1l1]@378

rattus_norvegicus	**Epb4.1l1**	FFRLV**SP**EPPP
drosophila_melanogaster	cora	FFRLM**TP**EPVS
drosophila_melanogaster	CG5022	FFTLPNSQSAA

ENSRNOP00000052108[Epb4.1l1]@443

rattus_norvegicus	**Epb4.1l1**	RPASV**S**ENHDA
drosophila_melanogaster	cora	EKEKVA-----
drosophila_melanogaster	CG5022	EPLGYGMPNGH

ENSRNOP00000052108[Epb4.1l1]@461

rattus_norvegicus	**Epb4.1l1**	EDDAE**S**GGRRS
drosophila_melanogaster	cora	SSTLDHRGDRN
drosophila_melanogaster	CG5022	MSDANGG----

ENSRNOP00000052108[Epb4.1l1]@475

rattus_norvegicus	**Epb4.1l1**	EGEVR**TP**TKIK
drosophila_melanogaster	cora	DAHSR**SP**IKNK
drosophila_melanogaster	CG5022	-------IQIS

ENSRNOP00000052108[Epb4.1l1]@544

rattus_norvegicus	**Epb4.1l1**	PSSPA**SP**SPKG
drosophila_melanogaster	cora	EKAAKAALAAG
drosophila_melanogaster	CG5022	-----------

ENSRNOP00000006443[Rtn4]@16

rattus_norvegicus	**Rtn4**	SSST-------D**SP**PRP-P
gallus_gallus	RTN4	SSS---------GPPRPQQ
pelodiscus_sinensis	RTN4	-------------------
anolis_carolinensis	RTN4	-------------------
xenopus_tropicalis	rtn4	SSSRPGGDPY-E**T**GERRQQ
drosophila_melanogaster	CG42853	-------------------
drosophila_melanogaster	Rtnl1	-------------------
drosophila_melanogaster	Rtnl2	-------------------
caenorhabditis_elegans	ret-1	NST---NEPTSEHEPKSLQ

ENSRNOP00000006443[Rtn4]@169

rattus_norvegicus	**Rtn4**	PKRRG**S**GSV------------------DE
gallus_gallus	RTN4	-----------------------------
pelodiscus_sinensis	RTN4	-----------------------------
anolis_carolinensis	RTN4	-----------------------------
xenopus_tropicalis	rtn4	-----**S**GSVA------YTFLAQRLPKEDE
drosophila_melanogaster	CG42853	-----------------------------
drosophila_melanogaster	Rtnl1	-----------------------------
drosophila_melanogaster	Rtnl2	-----------------------------
caenorhabditis_elegans	ret-1	IDLVE**S**GENAGIEQNAGAEQSQKEAVEET

ENSRNOP00000006443[Rtn4]@329

rattus_norvegicus	**Rtn4**	SA--ALH**SP**QESP
gallus_gallus	RTN4	---
pelodiscus_sinensis	RTN4	---EKIP
anolis_carolinensis	RTN4	----QGKP-------------------------------------DAEKFP
xenopus_tropicalis	rtn4	----SEKG-------------------------------------YVVEHP
drosophila_melanogaster	CG42853	---
drosophila_melanogaster	Rtnl1	---
drosophila_melanogaster	Rtnl2	---
caenorhabditis_elegans	ret-1	SSIKSEKNVIGLEDSGDTFRDTSGLAPAEDNEAEATITTDFVPLE**S**AGDIP

ENSRNOP00000006443[Rtn4]@343

rattus_norvegicus	**Rtn4**	EDR-----------------------------------VV**SP**EKTM
gallus_gallus	RTN4	--APE---
pelodiscus_sinensis	RTN4	Q-------------------------------------CM**SP**AVPV
anolis_carolinensis	RTN4	Q-------------------------------------DS**SP**DSPV
xenopus_tropicalis	rtn4	Q-------------------------------------ETISEEHA
drosophila_melanogaster	CG42853	--
drosophila_melanogaster	Rtnl1	--
drosophila_melanogaster	Rtnl2	--
caenorhabditis_elegans	ret-1	EIKEVASAPDVVGLEEYIIGNIPNAPVVNDDIPNVFTPEVANDETV

ENSRNOP00000006443[Rtn4]@425

rattus_norvegicus	**Rtn4**	RNEDA**S**FPSTP
gallus_gallus	RTN4	----------P
pelodiscus_sinensis	RTN4	SNEDL**S**FPSTP
anolis_carolinensis	RTN4	SNEDI**S**FPSTP
xenopus_tropicalis	rtn4	FSDDI**S**-**P**LTP
drosophila_melanogaster	CG42853	-----------
drosophila_melanogaster	Rtnl1	-TDDFE-----
drosophila_melanogaster	Rtnl2	-----------
caenorhabditis_elegans	ret-1	LEEDIM---SP

ENSRNOP00000006443[Rtn4]@488

rattus_norvegicus	**Rtn4**	ITEKT**SP**KTS--N
gallus_gallus	RTN4	-------------
pelodiscus_sinensis	RTN4	GTEQSDTITE---
anolis_carolinensis	RTN4	GTEPSGTQAE---
xenopus_tropicalis	rtn4	TSVGFGVKVATVN
drosophila_melanogaster	CG42853	-------------
drosophila_melanogaster	Rtnl1	-------------
drosophila_melanogaster	Rtnl2	-------------
caenorhabditis_elegans	ret-1	GKEEDDLENAN-D

ENSRNOP00000006443[Rtn4]@766

rattus_norvegicus	**Rtn4**	ERLSA**SP**QELG
gallus_gallus	RTN4	-----------
pelodiscus_sinensis	RTN4	EGLTDLQHSTG
anolis_carolinensis	RTN4	EKQEEMLLSLS
xenopus_tropicalis	rtn4	FDQKSEESSPS
drosophila_melanogaster	CG42853	-----------
drosophila_melanogaster	Rtnl1	--------PVA
drosophila_melanogaster	Rtnl2	-----------
caenorhabditis_elegans	ret-1	EEESAAIPEVQ

ENSRNOP00000019320[Map1a]@990

rattus_norvegicus	**Map1a**	TVKMA**SP**PPSG
gallus_gallus	MAP1A	TVKMA**SP**TQSG
anolis_carolinensis	MAP1A	TVKMA**SP**T---
xenopus_tropicalis	MAP1A	TVKMA**SP**THSG
drosophila_melanogaster	futsch	SVAEK**SP**L---
drosophila_melanogaster	dp	TLPSR**S**TDRTT

ENSRNOP00000019320[Map1a]@1280

rattus_norvegicus	**Map1a**	VDRKH**SP**---------------------------------GEIT
gallus_gallus	MAP1A	--
anolis_carolinensis	MAP1A	--
xenopus_tropicalis	MAP1A	--
drosophila_melanogaster	futsch	ARDDQ**SP**---------------------------------LESK
drosophila_melanogaster	dp	TSPSE**SP**ETPTTLPSDFITRPHSDQTTESTRDVPTTRPFEASTP

ENSRNOP00000019320[Map1a]@2026

rattus_norvegicus	**Map1a**	VEP--SI**TP**PAVP
gallus_gallus	MAP1A	HH---**SP**----
anolis_carolinensis	MAP1A	DE---**SP**PPLP
xenopus_tropicalis	MAP1A	---
drosophila_melanogaster	futsch	DSV---AEK**SP**-LAS
drosophila_melanogaster	dp	ENPCVPSPCGRNSQCRVVGETGVCSCLPNFVGRAPNCRPECTINTECPANLACINERCQDPCPGSCGFNAFCSVVNHSPICTCDSGYTGDPFAGCNPQPPAIP

ENSRNOP00000050877[Map2]@28

rattus_norvegicus	**Map2**	AAHPH**SP**EMKD
gallus_gallus	MAP2	ASHPH**SP**DIKE
pelodiscus_sinensis	MAP2	SSHPH**SP**EIKE
anolis_carolinensis	MAP2	SSHSHPPEIKE
xenopus_tropicalis	map2	SPLPHATDMKE
danio_rerio	map2	---PGAQGSSS
petromyzon_marinus	map2	-----------
drosophila_melanogaster	tau	-----------
caenorhabditis_elegans	ptl-1	---PQ**S**EPGSE

ENSRNOP00000050877[Map2]@657

rattus_norvegicus	**Map2**	PEEPS**SP**QERM
gallus_gallus	MAP2	SEEPS**SP**TERM
pelodiscus_sinensis	MAP2	PEEPS**SP**QERM
anolis_carolinensis	MAP2	PREPS**SP**KERI
xenopus_tropicalis	map2	LQASILPGDRL
danio_rerio	map2	EVQPDLPKSSA
petromyzon_marinus	map2	-----------
drosophila_melanogaster	tau	-----------
caenorhabditis_elegans	ptl-1	-----------

ENSRNOP00000050877[Map2]@1540

rattus_norvegicus	**Map2**	DGITK**SP**EK-RS
gallus_gallus	MAP2	DGVSK**SP**EK-RS
pelodiscus_sinensis	MAP2	DGVTK**SP**EK-RS
anolis_carolinensis	MAP2	DGIIK**SP**EK-RS
xenopus_tropicalis	map2	DGVSR**SP**EK-RS
danio_rerio	map2	DGRSR**SP**EK-RS
petromyzon_marinus	map2	------------
drosophila_melanogaster	tau	QLQQQPQQ-QQ
caenorhabditis_elegans	ptl-1	--ILRQPKPIPA

ENSRNOP00000050877[Map2]@1651

rattus_norvegicus	**Map2**	VALVR-**TP**PKS
gallus_gallus	MAP2	VALVR-**TP**PKS
pelodiscus_sinensis	MAP2	VALVR-**TP**PKS
anolis_carolinensis	MAP2	VALVR-**TP**PKS
xenopus_tropicalis	map2	VALVR-**TP**PKS
danio_rerio	map2	VALVR-**TP**PKS
petromyzon_marinus	map2	VALVR-**TP**PKS
drosophila_melanogaster	tau	EPVKK-VP---
caenorhabditis_elegans	ptl-1	----RM**TP**KVN

ENSRNOP00000006856[Mapt]@492

rattus_norvegicus	**Mapt**	TPSPK**T**---------**P**PG-------------------------SG
gallus_gallus	MAPT	PTAPK**T**---------**P**PG-SGRKE-------QKKPPPAAAKPEKA
pelodiscus_sinensis	MAPT	PTAPK**T**---------**P**PN-------------------------AG
anolis_carolinensis	MAPT	PTIPK**T**---------**P**PSLAAKKE-------QRRPPNTAAKSEKG
xenopus_tropicalis	mapt	SSIPK**T**---------**P**PS-AVRRD-------QRKPPPSGAKPDRA
drosophila_melanogaster	tau	PAPPQQQQP------PPH-QLQQQPQQQQLQQKPANARANQDQK
caenorhabditis_elegans	ptl-1	ESPPREQEPEKSGKSKPS-SPIPDAPTMEDIAPRELES-LNFSET

ENSRNOP00000006856[Mapt]@542

rattus_norvegicus	**Mapt**	VAVVR**TP**PKSP
gallus_gallus	MAPT	VAVVR**TP**PKSP
pelodiscus_sinensis	MAPT	VAVVR**TP**PKSP
anolis_carolinensis	MAPT	VAVVR**TP**PKSP
xenopus_tropicalis	mapt	IAVIR**TP**PKSP
drosophila_melanogaster	tau	----K**S**APKSR
caenorhabditis_elegans	ptl-1	QRAIS**TP**RQTA

ENSRNOP00000006856[Mapt]@707

rattus_norvegicus	**Mapt**	EIVYK**SP**VVSG
gallus_gallus	MAPT	EIVYK**SP**TISG
pelodiscus_sinensis	MAPT	EIVYK**SP**TVSG
anolis_carolinensis	MAPT	EIVYK**SP**TVSG
xenopus_tropicalis	mapt	EIVYK**SP**GQSG
drosophila_melanogaster	tau	KKIFDDKDY--
caenorhabditis_elegans	ptl-1	TIIDKKKDFAK

ENSRNOP00000006856[Mapt]@715

rattus_norvegicus	**Mapt**	VSGDT**SP**RHLS
gallus_gallus	MAPT	ISGDA**SP**RRLS
pelodiscus_sinensis	MAPT	VSGDA**SP**RRLS
anolis_carolinensis	MAPT	VSGDA**SP**RRLS
xenopus_tropicalis	mapt	QSGET**SP**RRLS
drosophila_melanogaster	tau	Y--------LK
caenorhabditis_elegans	ptl-1	FAKRQ**S**VSQRS

ENSRNOP00000021491[Add2]@594

rattus_norvegicus	**Add2**	TEEPG**SP**-------VKST
gallus_gallus	ADD2	TQ--A**SP**-------PKSP
pelodiscus_sinensis	ADD2	VE--A**SP**-------VKSP
anolis_carolinensis	ADD2	LQ--AAPGEKDAGLKEDP
xenopus_tropicalis	add2	ER--EQPEEDSQPSLVTP
danio_rerio	add2	QQ--DGVEEVVNDTGSSP
drosophila_melanogaster	hts	SE--SEAV-LQAGTKKYP
caenorhabditis_elegans	add-1	SQ--SAPVSARSGYSQYD
caenorhabditis_elegans	add-2	------------------

ENSRNOP00000021491[Add2]@702

rattus_norvegicus	**Add2**	PS---KSP**S**KKK---KK
gallus_gallus	ADD2	PS---KSP**S**KKK---KK
pelodiscus_sinensis	ADD2	PS---KSP**S**KKK---KK
anolis_carolinensis	ADD2	PS---KSP**S**KKK---KK
xenopus_tropicalis	add2	PT---KSP**S**KKK---KK
danio_rerio	add2	PS---KSP**S**KKK---KK
drosophila_melanogaster	hts	PTPRMSRSWSEDQQSEDDDQKKNEAGERGYIDNDTPIDRVISSTTFVCRRSP**T**RKRNFIQENIRNASRPRIAAGSIRKHKSNIVTSPRAKSSPSSVYTFQCGQRDSGARLWVALPTSPRGQNDEKSSTAARALYTVRGNPPSPFAKR
caenorhabditis_elegans	add-1	PT---KDEKKKK---KK
caenorhabditis_elegans	add-2	PN---

ENSRNOP00000027691[Apc]@1772

rattus_norvegicus	**Apc**	KK-------------KPT**SP**VKPM
xenopus_tropicalis	apc	KN-------------KPT**SP**VKPM
danio_rerio	apc	KK-------------KPT**SP**VKPM
petromyzon_marinus	apc	------------------------
drosophila_melanogaster	Apc	QKAHATSNPQQQSSTHPS**S**HILPN
caenorhabditis_elegans	apr-1	------------------------

ENSRNOP00000065853[Nefm]@502

rattus_norvegicus	**Nefm**	PEVEK**SP**VKSP
gallus_gallus	NEFM	AEEEAVEEEAV
pelodiscus_sinensis	NEFM	VAEKKAPVKAA
anolis_carolinensis	NEFM	VAEEKAEVEAA
xenopus_tropicalis	nefm	VAAVE**S**SVQAA
drosophila_melanogaster	LamC	NAAAKGDLEII
drosophila_melanogaster	Lam	SASAKGNVEIK
caenorhabditis_elegans	ifc-1	ESSYSYSSSNN
caenorhabditis_elegans	ifb-1	QRSAKGNVTIS
caenorhabditis_elegans	ifa-3	QRSAKGNVSIK
caenorhabditis_elegans	ifc-2	HSWYLGTISIN
caenorhabditis_elegans	ifb-2	KRHAKGNVSIV
caenorhabditis_elegans	ifa-1	QRSAKGNVSIH
caenorhabditis_elegans	ifa-4	SRSAKGNIAIQ
caenorhabditis_elegans	ifd-1	RSYGKGDVKII
caenorhabditis_elegans	ifd-2	-----------
caenorhabditis_elegans	mua-6	QRSAKGNVAIK
caenorhabditis_elegans	lmn-1	NKETVGPVGID

ENSRNOP00000065853[Nefm]@603

rattus_norvegicus	**Nefm**	PEKAK**SP**---VPKS
gallus_gallus	NEFM	SPPAK**SP**PKSPPKS
pelodiscus_sinensis	NEFM	--------------
anolis_carolinensis	NEFM	VEKAKAS---PPKS
xenopus_tropicalis	nefm	IVREQAA-------
drosophila_melanogaster	LamC	WSVDAGTAHDPPNN
drosophila_melanogaster	Lam	WSADTKASHEPPSS
caenorhabditis_elegans	mua-6	FARGQ-GIANPPEV
caenorhabditis_elegans	ifc-1	--------------
caenorhabditis_elegans	ifb-1	YARDQGGINNPPES
caenorhabditis_elegans	ifa-3	FARGN-GVANPPEV
caenorhabditis_elegans	ifc-2	NHRHGKYL----GQ
caenorhabditis_elegans	ifd-1	HSLK-**SP**NLM--DD
caenorhabditis_elegans	ifb-2	YGRNSGGINSPPDS
caenorhabditis_elegans	ifa-1	FARNQ-GVASPPDQ
caenorhabditis_elegans	ifa-4	FARGQ-GVHSPPDS
caenorhabditis_elegans	ifd-2	------------DS
caenorhabditis_elegans	lmn-1	WSADAGAVHHPPEV

ENSRNOP00000065853[Nefm]@608

rattus_norvegicus	**Nefm**	SP---VPK**SP**VEEV
gallus_gallus	NEFM	SPPKSPPK**SP**VTEQ
pelodiscus_sinensis	NEFM	--------------
anolis_carolinensis	NEFM	AS---PPK**SP**VGD-
xenopus_tropicalis	nefm	AA--------VQE-
drosophila_melanogaster	LamC	GTAHDPPNNLVMK-
drosophila_melanogaster	Lam	KASHEPPS**S**LVMKS
caenorhabditis_elegans	ifc-1	--------------
caenorhabditis_elegans	ifb-1	GGINNPPE**S**LVFDG
caenorhabditis_elegans	ifa-3	-GVANPPEVLVFEG
caenorhabditis_elegans	ifc-2	KYL----GQFFMDV
caenorhabditis_elegans	ifb-2	GGINSPPD**S**IVMES
caenorhabditis_elegans	ifa-1	-GVASPPDQLVYDA
caenorhabditis_elegans	ifa-4	-GVHSPPD**S**LVYDL
caenorhabditis_elegans	ifd-1	PNLM---DDIVATQ
caenorhabditis_elegans	ifd-2	-------D**S**LL---
caenorhabditis_elegans	mua-6	-GIANPPEVLIFEG
caenorhabditis_elegans	lmn-1	GAVHHPPEVYVMKK

ENSRNOP00000065853[Nefm]@642

rattus_norvegicus	**Nefm**	EVAKE**SP**KEE-K
gallus_gallus	NEFM	AASPEKPATP-K
pelodiscus_sinensis	NEFM	TGSPE-------
anolis_carolinensis	NEFM	AGTPEKASSPVK
xenopus_tropicalis	nefm	AGEDKQAKTP--
drosophila_melanogaster	LamC	------------
drosophila_melanogaster	Lam	------------
caenorhabditis_elegans	ifc-1	------------
caenorhabditis_elegans	ifb-1	------------
caenorhabditis_elegans	ifa-3	------------
caenorhabditis_elegans	ifc-2	------------
caenorhabditis_elegans	ifb-2	------------
caenorhabditis_elegans	ifa-1	------------
caenorhabditis_elegans	ifa-4	------------
caenorhabditis_elegans	ifd-1	------------
caenorhabditis_elegans	ifd-2	------------
caenorhabditis_elegans	mua-6	------------
caenorhabditis_elegans	lmn-1	------------

ENSRNOP00000065853[Nefm]@766

rattus_norvegicus	**Nefm**	NGL-DV**SP**AEEK
gallus_gallus	NEFM	NGL-DV**SP**VDEK
pelodiscus_sinensis	NEFM	------------
anolis_carolinensis	NEFM	NGL-DV**SP**VEEK
xenopus_tropicalis	nefm	NGLEDD**SP**SEDK
drosophila_melanogaster	LamC	----N-------
drosophila_melanogaster	Lam	----N-------
caenorhabditis_elegans	ifc-1	------------
caenorhabditis_elegans	ifb-1	----N-------
caenorhabditis_elegans	ifa-3	----N-------
caenorhabditis_elegans	ifc-2	----N-------
caenorhabditis_elegans	ifb-2	----N-------
caenorhabditis_elegans	ifa-1	----N-------
caenorhabditis_elegans	ifa-4	----N-------
caenorhabditis_elegans	ifd-1	----D-------
caenorhabditis_elegans	ifd-2	------------
caenorhabditis_elegans	mua-6	----N-------
caenorhabditis_elegans	lmn-1	----D-------

ENSRNOP00000022574[Stmn1]@25

rattus_norvegicus	**Stmn1**	FELIL**S**-**P**RSKE
rattus_norvegicus	Stmn1	FELILN-PWSKQ
gallus_gallus	STMN1	FELILG-PRSKE
pelodiscus_sinensis	STMN1	FELIL**S**-**P**RSKE
anolis_carolinensis	STMN1	FELILG-PPSKD
xenopus_tropicalis	stmn1	------------
drosophila_melanogaster	stai	YEVILAEPAPNV

ENSRNOP00000022574[Stmn1]@38

rattus_norvegicus	**Stmn1**	PEFPL**SP**P----KKK
rattus_norvegicus	Stmn1	PEFPLPHPPSLAKKK
gallus_gallus	STMN1	PEFPL**SP**P----KKK
pelodiscus_sinensis	STMN1	PEFPL**SP**P----KKK
anolis_carolinensis	STMN1	PEFPL**SP**P----KKK
xenopus_tropicalis	stmn1	---------------
drosophila_melanogaster	stai	PKRPV**TP**------GK

ENSRNOP00000022574[Stmn1]@46

rattus_norvegicus	**Stmn1**	KKKDL**S**LEEIQ
rattus_norvegicus	Stmn1	KKKDL**S**LEEIQ
gallus_gallus	STMN1	KKKDL**S**LEEIQ
pelodiscus_sinensis	STMN1	KKKDL**S**LEEIK
anolis_carolinensis	STMN1	KKKDL**S**LEEIQ
xenopus_tropicalis	stmn1	-----------
drosophila_melanogaster	stai	-GKNV**S**VEEIE

ENSRNOP00000046963[Stmn1]@50

rattus_norvegicus	Stmn1	KKKDLSLEEIQ
rattus_norvegicus	**Stmn1**	KKKDLSLEEIQ
gallus_gallus	STMN1	KKKDLSLEEIQ
pelodiscus_sinensis	STMN1	KKKDLSLEEIK
anolis_carolinensis	STMN1	KKKDLSLEEIQ
xenopus_tropicalis	stmn1	-----------
drosophila_melanogaster	stai	-GKNVSVEEIE

ENSRNOP00000015720[Stmn2]@62

rattus_norvegicus	**Stmn2**	ILKPP**SP**ISEA
gallus_gallus	STMN2	ILKPP**SP**VSEA
pelodiscus_sinensis	STMN2	ILKPP**SP**VSEA
anolis_carolinensis	STMN2	ILKPP**SP**VSEA
xenopus_tropicalis	stmn2	VIKLQWPGDKG
drosophila_melanogaster	stai	ILAEPAPNVAV

ENSRNOP00000015720[Stmn2]@80

rattus_norvegicus	**Stmn2**	KKKDL**S**LEEIQ
gallus_gallus	STMN2	KKKEL**S**LEEIQ
pelodiscus_sinensis	STMN2	KKKEL**S**REEIQ
anolis_carolinensis	STMN2	KKKDL**S**LDEIQ
xenopus_tropicalis	stmn2	RGKCI**S**GDSHN
drosophila_melanogaster	stai	-GKNV**S**VEEIE

ENSRNOP00000016070[Srcin1]@260

rattus_norvegicus	**Srcin1**	MGMLK**SP**NTAI
gallus_gallus	SRCIN1	MGMLK**SP**NTAI
anolis_carolinensis	SRCIN1	MGMLK**SP**NTAI
xenopus_tropicalis	srcin1	MGMLK**S**HNTAI
danio_rerio	SRCIN1 (1 of 2)	MGALR**SP**GTAL
danio_rerio	SRCIN1 (2 of 2)	AGMLK**SP**NTAI
drosophila_melanogaster	CG32809	MSYLEGPNVKI
caenorhabditis_elegans	Y71H2AM.15	RSFVEQPHVKI

ENSRNOP00000018072[Add1]@12

rattus_norvegicus	**Add1**	AAVVT**SP**PPT-T
gallus_gallus	ADD1	VAVVT**SP**PPT-T
pelodiscus_sinensis	ADD1	ATVVT**SP**PPT-T
anolis_carolinensis	ADD1	AEVVT**SP**-LT-S
xenopus_tropicalis	add1	AGVVT**TP**PPT-N
danio_rerio	add1	AGVVTAPPPT-N
petromyzon_marinus	add1	GGVVAVAPVA-A
drosophila_melanogaster	hts	-----------V
caenorhabditis_elegans	add-1	------------
caenorhabditis_elegans	add-2	QKILNIPGTSFS

ENSRNOP00000018072[Add1]@355

rattus_norvegicus	**Add1**	KAKSR**SP**G----------------TPA
gallus_gallus	ADD1	KAKSR**S**SE----------------SPS
pelodiscus_sinensis	ADD1	KAKSR**SP**E----------------SPA
anolis_carolinensis	ADD1	KAKSHCNE----------------SPT
xenopus_tropicalis	add1	K-KSR**SP**E----------------TPS
danio_rerio	add1	KSRPRHLE-----------------PA
petromyzon_marinus	add1	RVRTQGIV----------------THG
drosophila_melanogaster	hts	KAIYEQSRRPPEDLEKKFAAVAAAEDG
caenorhabditis_elegans	add-1	QRAFRNSR----------------NTN
caenorhabditis_elegans	add-2	KQVYKVAS---------------QGGG

ENSRNOP00000018072[Add1]@586

rattus_norvegicus	**Add1**	RKQKG**S**EENL--D
gallus_gallus	ADD1	RKQKG**S**EEPS--E
pelodiscus_sinensis	ADD1	RKQKG**S**EEPS--E
anolis_carolinensis	ADD1	RKQRQ**T**EEDS--E
xenopus_tropicalis	add1	RKQKGAEENC--G
danio_rerio	add1	LKQKGGEDEAQVE
petromyzon_marinus	add1	-------------
drosophila_melanogaster	hts	RKKKSVHGEY--T
caenorhabditis_elegans	add-1	AKN----------
caenorhabditis_elegans	add-2	-------------

ENSRNOP00000018072[Add1]@610

rattus_norvegicus	**Add1**	SAVP-----N**TP**PSTP
gallus_gallus	ADD1	TSA------C**TP**PSTP
pelodiscus_sinensis	ADD1	TSA------R**TP**PSTP
anolis_carolinensis	ADD1	TVA------C**TP**PSTP
xenopus_tropicalis	add1	KEAPV-EHIR**TP**PSTP
danio_rerio	add1	EEGKVPSSTC**TP**PSSP
petromyzon_marinus	add1	----------------
drosophila_melanogaster	hts	TAASVGQ--CVQPEVC
caenorhabditis_elegans	add-1	SQSVVFD--CDHPSPM
caenorhabditis_elegans	add-2	----------------

ENSRNOP00000011604[Nefh]@670

rattus_norvegicus	**Nefh**	PAEAK**SP**VEVK
gallus_gallus	NEFH	-----------
pelodiscus_sinensis	NEFH	PEPKD**T**KETPK
anolis_carolinensis	NEFH	TEDNQKKAT-K
xenopus_tropicalis	nefh	KTEPVKKDEGK
drosophila_melanogaster	LamC	-----------
drosophila_melanogaster	Lam	-----------
caenorhabditis_elegans	ifc-1	-----------
caenorhabditis_elegans	ifb-1	-----------
caenorhabditis_elegans	ifa-3	-----------
caenorhabditis_elegans	ifc-2	-----------
caenorhabditis_elegans	ifb-2	-----------
caenorhabditis_elegans	ifa-1	-----------
caenorhabditis_elegans	ifa-4	-----------
caenorhabditis_elegans	ifd-1	-----------
caenorhabditis_elegans	ifd-2	-----------
caenorhabditis_elegans	mua-6	-----------
caenorhabditis_elegans	lmn-1	-----------

ENSRNOP00000011604[Nefh]@748

rattus_norvegicus	**Nefh**	PAEAK**SP**VEVK
gallus_gallus	NEFH	PA-------VK
pelodiscus_sinensis	NEFH	PKAETPKEAPK
anolis_carolinensis	NEFH	VKVEKKKGEEK
xenopus_tropicalis	nefh	PSTKVVPTEEE
drosophila_melanogaster	LamC	-----------
drosophila_melanogaster	Lam	-----------
caenorhabditis_elegans	ifc-1	-----------
caenorhabditis_elegans	ifb-1	-----------
caenorhabditis_elegans	ifa-3	-----------
caenorhabditis_elegans	ifc-2	-----------
caenorhabditis_elegans	ifb-2	-----------
caenorhabditis_elegans	ifa-1	-----------
caenorhabditis_elegans	ifa-4	-----------
caenorhabditis_elegans	ifd-1	-----------
caenorhabditis_elegans	ifd-2	-----------
caenorhabditis_elegans	mua-6	-----------
caenorhabditis_elegans	lmn-1	-----------

ENSRNOP00000022849[Epb41l3]@490

rattus_norvegicus	**Epb41l3**	LITTV**TP**EKKA
gallus_gallus	EPB41L3	LITTV**TP**EKKA
pelodiscus_sinensis	EPB41L3	LITTV**TP**EKKP
anolis_carolinensis	EPB41L3	LITTV**TP**EKKA
xenopus_tropicalis	epb41l3	LITTV**T**QRNNN
drosophila_melanogaster	cora	ADKEAKLREKK
drosophila_melanogaster	CG5022	NIDGVNYLAS-

ENSRNOP00000064845[Rtn1]@469

rattus_norvegicus	**RTN1**	KREQ--D**SP**P--MKP
gallus_gallus	RTN1	KREQ--D**SP**L--MKP
pelodiscus_sinensis	RTN1	KREQ--D**SP**L--MKP
anolis_carolinensis	RTN1	KREH--D**SP**L--MKP
xenopus_tropicalis	rtn1	KRDQ--D**SP**M--MKP
drosophila_melanogaster	CG42853	---
drosophila_melanogaster	Rtnl1	KAPT---IAAPV--VEP
drosophila_melanogaster	Rtnl2	---
caenorhabditis_elegans	ret-1	EKDNESLEAPEIINEPIRRVLVETKIMGPGKSLNEDNDDDDDGSECLDSIGDLSERTIQRFNTSIDDPSIRRDSFSSISSFGDRQKFRTAIENIRQDLLPFQSSVSQYLRSSPNPSQQLLVTNLSMD**SP**SDLSPNAPPVGFENTAQFLEKLQQEDRPSAEGSIDSSGFEKVDHEGLDEFAAPPVHDPMQKSVFGSLGSDDMKP

ENSRNOP00000053011[Map4]@618

rattus_norvegicus	**MAP4**	PVDEG**SP**LEKL
gallus_gallus	MAP4	ARSSL**SP**KQDS
pelodiscus_sinensis	MAP4	PPQEK**SP**LKPT
anolis_carolinensis	MAP4	PMQPG**TP**LLET
xenopus_tropicalis	map4	EHKAA**S**LVEP-
danio_rerio	MAP4 (1 of 3)	PDSKTKPSAAS
danio_rerio	MAP4 (2 of 3)	-----------
danio_rerio	MAP4 (3 of 3)	PEKKTKPVAGL
drosophila_melanogaster	tau	-----------
caenorhabditis_elegans	ptl-1	PEVAVEP----

ENSRNOP00000053011[Map4]@687

rattus_norvegicus	**MAP4**	AEKRT**SP**-----SKPS
gallus_gallus	MAP4	PDKRT**S**L------SKP
pelodiscus_sinensis	MAP4	PEKRT**S**V-----SKPL
anolis_carolinensis	MAP4	LDKRT**SP**-----SKPP
xenopus_tropicalis	map4	PVKSPDKKPAALKQTP
danio_rerio	MAP4 (1 of 3)	PSSTVE-------NKA
danio_rerio	MAP4 (2 of 3)	----------------
danio_rerio	MAP4 (3 of 3)	ATATARP--PTATTAA
drosophila_melanogaster	tau	----------------
caenorhabditis_elegans	ptl-1	-EKEEEV---VVESPP

ENSRNOP00000019569[Dbn1]@142

rattus_norvegicus	**Dbn1**	LARLS**SP**VLHR
gallus_gallus	DBN1	LARVS**SP**VLHR
anolis_carolinensis	DBN1	LARIA**SP**VLHR
xenopus_tropicalis	dbn1	SGRLS**SP**VLQR
danio_rerio	dbn1	TVPVA**SP**VLSR
drosophila_melanogaster	CG10083	SSAYS---FKE
drosophila_melanogaster	Cortactin	VGKHA**S**QKDYS
caenorhabditis_elegans	K08E3.4	PAANANAIVES

ENSRNOP00000019569[Dbn1]@383

rattus_norvegicus	**Dbn1**	PIPTR**SP**SDSS
gallus_gallus	DBN1	C----**SP**CESS
anolis_carolinensis	DBN1	-----**SP**CDSS
xenopus_tropicalis	dbn1	------------
danio_rerio	dbn1	------------
drosophila_melanogaster	CG10083	-----KPA---
drosophila_melanogaster	Cortactin	-----KGS---
caenorhabditis_elegans	K08E3.4	-----APT---

ENSRNOP00000048424[Sgip1]@275

rattus_norvegicus	**Sgip1**	PFPTG**TP**PPLP
gallus_gallus	SGIP1	PFPIG**TP**PPLP
pelodiscus_sinensis	SGIP1	PFPTG**S**KLIIS
anolis_carolinensis	SGIP1	PFPSG**TP**PPLP
xenopus_tropicalis	sgip1	PYPTG**TP**PPIP
danio_rerio	SGIP1	-------PPLP
drosophila_melanogaster	CG8176	STPTA**S**VHPYA
caenorhabditis_elegans	fcho-1	RQPLR**S**HH---

ENSRNOP00000008210[Sptbn1]@2096

rattus_norvegicus	**Sptbn1**	KRRPP**SP**EPSA
gallus_gallus	SPTBN1	KRKPP**TP**EPSP
pelodiscus_sinensis	SPTBN1	KRQPP**SP**EPSP
anolis_carolinensis	SPTBN1	KRQPP**SP**EPSP
xenopus_tropicalis	SPTBN1	KRKPP**TP**ELSP
danio_rerio	SPTBN1 (1 of 2)	-----------
petromyzon_marinus	SPTBN1 (2 of 2)	KQQKLQAELDE
petromyzon_marinus	SPTBN1 (1 of 2)	-----------
drosophila_melanogaster	beta-Spec	RQRIK------
caenorhabditis_elegans	unc-70	RRGPA------

ENSRNOP00000008210[Sptbn1]@2131

rattus_norvegicus	**Sptbn1**	PAEQG**SP**RMAG
gallus_gallus	SPTBN1	PSDQE**SP**RVAE
pelodiscus_sinensis	SPTBN1	PSDQE**SP**RMAD
anolis_carolinensis	SPTBN1	PPDQE**SP**RMGE
xenopus_tropicalis	SPTBN1	PSDQE**SP**RMAE
danio_rerio	SPTBN1 (1 of 2)	-----------
petromyzon_marinus	SPTBN1 (2 of 2)	SSDQE**SP**RQGS
petromyzon_marinus	SPTBN1 (1 of 2)	-----------
drosophila_melanogaster	beta-Spec	EAAEQAKREAE
caenorhabditis_elegans	unc-70	PAAET**S**-----

ENSRNOP00000015454[Rims1]@895

rattus_norvegicus	Rims1	PLPQP**SP**------FMPR
gallus_gallus	RIMS1	PLPQP**SP**------FMPR
pelodiscus_sinensis	RIMS1	PLPQP**SP**------FMPR
anolis_carolinensis	RIMS1	PLPQP**SP**------FMPR
xenopus_tropicalis	rims1	PLPQP**SP**------FMPR
drosophila_melanogaster	CG43955	SISK-**SP**S-----VSPR
drosophila_melanogaster	Rim	LLRDEGSDVDGLILTPT
caenorhabditis_elegans	unc-10	PL---------------

ENSRNOP00000015454[Rims1]@1078

rattus_norvegicus	**Rims1**	DTSLH**SP**ERE--R
gallus_gallus	RIMS1	DTSLH**SP**ERERGRWSPSLERRRPTSPRIHIQHASPEDDRTR-TLEFCIPKQDSVDHLSAKPGAA-QR
pelodiscus_sinensis	RIMS1	DTSLH**SP**ERE----------------------------RTR-TLESCIAKQDSINHLSAKPGAT-QR
anolis_carolinensis	RIMS1	DTSLH**SP**EQERHRWSPSLDRRRPTSPRIHIQHASPEDDRTTPPLESCIPKQDTLNFLSANPEET-QR
xenopus_tropicalis	rims1	DTSLH**SP**ERE--R
drosophila_melanogaster	Rim	TSILKKPKLERRRLFHEGQSRSLDYDDLHTK--SWGRRRIR-YEDEVMPSSGDYPYPHSNPYSSYSP
drosophila_melanogaster	CG43955	DEILK**S**LKAVRGEL---
caenorhabditis_elegans	unc-10	RSATALSQMEREDMYDPTRKHRDD-----------------------------------NEYSM---

ENSRNOP00000063889[Kidins220]@1547

rattus_norvegicus	**Kidins220**	ERVCK**SP**EHSA
gallus_gallus	KIDINS220	DKQPK**SP**EHGS
pelodiscus_sinensis	KIDINS220	DKQPK**SP**EHGS
anolis_carolinensis	KIDINS220	EKQPK**SP**EHGS
xenopus_tropicalis	kidins220	DK--KAGELAS
drosophila_melanogaster	Fem-1	-----------
drosophila_melanogaster	CG42672	----------S
drosophila_melanogaster	m-cup	-----------
drosophila_melanogaster	CG6966	-----------
caenorhabditis_elegans	fem-1	-----------
caenorhabditis_elegans	kdin-1	---------GA
caenorhabditis_elegans	Y39C12A.1	-----------
caenorhabditis_elegans	C01H6.2	-----------

ENSRNOP00000044022[Scrib]@506

rattus_norvegicus	**Scrib**	DPSPP--**SP**SEEE
gallus_gallus	SCRIB	GTDLN--**S**SSSEE
pelodiscus_sinensis	SCRIB	DVNPD--**SP**DSEE
xenopus_tropicalis	scrib	EAT----CASDEE
danio_rerio	scrib	DEDLE--**SP**DAEE
drosophila_melanogaster	scrib	EENGTKLAETPTE
caenorhabditis_elegans	rsu-1	-------------
caenorhabditis_elegans	let-413	-------HEIDQP

ENSRNOP00000020123[Apba1]@83

rattus_norvegicus	**Apba1**	LARSA**S**TESGF
gallus_gallus	APBA1	LARSA**S**TESGF
pelodiscus_sinensis	APBA1	LARSA**S**TESGF
anolis_carolinensis	APBA1	LARSA**S**TESGF
xenopus_tropicalis	APBA1	LARSA**S**TESGF
drosophila_melanogaster	X11Lbeta	AQ--APPTAVA
drosophila_melanogaster	X11L	PQRVSPTTSVA
caenorhabditis_elegans	lin-10	SQASS**S**HRSED

ENSRNOP00000020123[Apba1]@316

rattus_norvegicus	**Apba1**	GGHPD**SP**GLPA
gallus_gallus	APBA1	GGGHE**S**NNSQL
pelodiscus_sinensis	APBA1	RGTLE**S**-NGQL
anolis_carolinensis	APBA1	NVRPE**S**-NSQL
xenopus_tropicalis	APBA1	GGSHV**T**-NGNL
drosophila_melanogaster	X11Lbeta	APAPHR----K
drosophila_melanogaster	X11L	GGTHTE----K
caenorhabditis_elegans	lin-10	NGTAPL----V

ENSRNOP00000042382[Sptan1]@1031

rattus_norvegicus	**Sptan1**	PAQSA**S**RE------NLL
gallus_gallus	SPTAN1	PAQSA**S**RE------NLL
pelodiscus_sinensis	SPTAN1	PAQSA**S**RE------NLL
xenopus_tropicalis	sptan1	PAQSA**S**RE------NLL
drosophila_melanogaster	alpha-Spec	AGLSA**S**QQ------NLV
drosophila_melanogaster	Msp-300	PDDSS**S**NQEGELPIHVD
caenorhabditis_elegans	spc-1	PG--TAQQ------HAQ
caenorhabditis_elegans	anc-1	PERSILDTIAKSDIPAL

ENSRNOP00000009556[Hsp90aa1]@263

rattus_norvegicus	**Hsp90aa1**	IEDVG**S**DEEEE
gallus_gallus	HSP90AA1	IEDVG**S**D-EEE
pelodiscus_sinensis	HSP90AA1	IEDVG**S**D-EEE
anolis_carolinensis	HSP90AA1	-----------
drosophila_melanogaster	Hsp83	IEDVGED-EDA
caenorhabditis_elegans	daf-21	VENVAD-----

ENSRNOP00000007972[Ctnna2]@623

rattus_norvegicus	**Ctnna2**	VRSRT**S**VQTED
gallus_gallus	CTNNA2	VRSRT**S**VQTED
danio_rerio	ctnna2	ARSRT**S**IQTED
drosophila_melanogaster	alpha-Cat	TRSRS**S**AHTGD
drosophila_melanogaster	Vinc	----APPQPPP
drosophila_melanogaster	alpha-catenin-related	-----------
caenorhabditis_elegans	deb-1	SISRA**SP**YNPP
caenorhabditis_elegans	hmp-1	AANADANRT--
caenorhabditis_elegans	ctn-1	-----------

ENSRNOP00000007972[Ctnna2]@908

rattus_norvegicus	**Ctnna2**	QKKHI**SP**VQAL
gallus_gallus	CTNNA2	QKKHI**SP**VQAL
danio_rerio	ctnna2	QKKHI**SP**VQAL
drosophila_melanogaster	alpha-Cat	QKKVQNPIHAL
drosophila_melanogaster	Vinc	---------PW
drosophila_melanogaster	alpha-catenin-related	-----------
caenorhabditis_elegans	deb-1	---------MW
caenorhabditis_elegans	hmp-1	ERRPLQPAKVL
caenorhabditis_elegans	ctn-1	-QQLIKHTHLV

ENSRNOP00000008041[Ctnna1]@657

rattus_norvegicus	**Ctnna1**	VRSRT**S**VQTED
gallus_gallus	CTNNA1	-----------
pelodiscus_sinensis	CTNNA1	VRSRT**S**VQTED
anolis_carolinensis	CTNNA1	VRSRT**S**VQTED
xenopus_tropicalis	ctnna1	VRSRT**S**VQTED
danio_rerio	ctnna1	SRSRT**S**VQTED
drosophila_melanogaster	alpha-Cat	TRSRS**S**AHTGD
drosophila_melanogaster	Vinc	PVRPPPPETDD
drosophila_melanogaster	alpha-catenin-related	-----------
caenorhabditis_elegans	deb-1	PPRPPPPPEYD
caenorhabditis_elegans	hmp-1	AANADANRTIS
caenorhabditis_elegans	ctn-1	-----------

ENSRNOP00000041065[Macf1]@2429

rattus_norvegicus	**Macf1**	MDASL**SP**TKTE
pelodiscus_sinensis	MACF1	-----------
anolis_carolinensis	MACF1	-----------
xenopus_tropicalis	macf1	VN--V**SP**TKSE
danio_rerio	macf1	GQ-TA**SP**SRPE
petromyzon_marinus	macf1	WS--GAPSEGS
drosophila_melanogaster	shot	------PKESV
caenorhabditis_elegans	vab-10	GF--PVPATKE

ENSRNOP00000041065[Macf1]@2585

rattus_norvegicus	**Macf1**	GPGDV**SP**SASQ
pelodiscus_sinensis	MACF1	-----------
anolis_carolinensis	MACF1	-----------
xenopus_tropicalis	macf1	GPGEE**SP**STCQ
danio_rerio	macf1	LTGDERSRDEQ
petromyzon_marinus	macf1	KGGENREANER
drosophila_melanogaster	shot	HLAGTPDAQA-
caenorhabditis_elegans	vab-10	DGGANVEE---

ENSRNOP00000018960[Eps15l1]@108

rattus_norvegicus	**Eps15l1**	F----------------------HDTS**SP**LM----------------AT
gallus_gallus	EPS15L1	F----------------------HDTS**SP**LL----------------IT
pelodiscus_sinensis	EPS15L1	F----------------------HDTS**SP**LL----------------VT
anolis_carolinensis	EPS15L1	---
xenopus_tropicalis	eps15l1	F--------------------FQHDTS**SP**LL----------------IT
danio_rerio	EPS15L1 (2 of 2)	F----------------------RDAG**SP**SL---------------NIT
drosophila_melanogaster	Eps-15	L--------------------GELPKTMP----------SRIQTVPVAS
drosophila_melanogaster	Dap160	---
caenorhabditis_elegans	itsn-1	---
caenorhabditis_elegans	ehs-1	FAPPVPPANMQHHFQPSFPSVGRAPPVPPPPHPSHPYSISSQQSSPAHP
caenorhabditis_elegans	reps-1	R----------------------LKHT**SP**-------------------A

ENSRNOP00000018960[Eps15l1]@229

rattus_norvegicus	**Eps15l1**	PVLPA**SP**-PPKD
gallus_gallus	EPS15L1	PVLPA**SP**-PPKD
pelodiscus_sinensis	EPS15L1	PVLPA**SP**-PPKD
anolis_carolinensis	EPS15L1	------------
xenopus_tropicalis	eps15l1	SVLPS**SP**-PPKD
danio_rerio	EPS15L1 (2 of 2)	PVLPS**SP**FLLKE
drosophila_melanogaster	Eps-15	PPLPVAVPP---
drosophila_melanogaster	Dap160	PVVPVVAPPVAV
caenorhabditis_elegans	itsn-1	--------PPAI
caenorhabditis_elegans	ehs-1	PPQP--------
caenorhabditis_elegans	reps-1	PVKNWANQPIIK

ENSRNOP00000018960[Eps15l1]@255

rattus_norvegicus	**Eps15l1**	STGSL**SP**---KHSV
gallus_gallus	EPS15L1	STGSL**SP**---KHSI
pelodiscus_sinensis	EPS15L1	STGSL**SP**---KHSI
anolis_carolinensis	EPS15L1	--------------
xenopus_tropicalis	eps15l1	SAGSL**SP**---KHSS
danio_rerio	EPS15L1 (2 of 2)	SSTNL**SP**---SNSF
drosophila_melanogaster	Eps-15	-------------L
drosophila_melanogaster	Dap160	TPPTSNPPSRHTSI
caenorhabditis_elegans	itsn-1	VSIAG**TP**SSRHNSI
caenorhabditis_elegans	ehs-1	TSGAS**TP**ISASHSI
caenorhabditis_elegans	reps-1	TAIKH**S**ALLALKSP

ENSRNOP00000030821[Snap91]@314

rattus_norvegicus	**Snap91**	ATTVT**SP**NS-----TP
gallus_gallus	SNAP91	ATTVT**SP**NT-----TP
pelodiscus_sinensis	SNAP91	ATTVT**SP**NS-----TP
anolis_carolinensis	SNAP91	ATTVT**SP**NS-----TP
xenopus_tropicalis	snap91	ATTVT**SP**NA-----TP
danio_rerio	snap91	TNNG-**TP**AG-----TP
danio_rerio	SNAP91 (1 of 2)	TNNV-**SP**TS-----TP
drosophila_melanogaster	lap	QRNVK**S**AVSALSSTSS
caenorhabditis_elegans	unc-11	PHQFT**T**GFAFSQQPQP

ENSRNOP00000019748[Dclk1]@30

rattus_norvegicus	**Dclk1**	SPSPT**SP**GSLR
gallus_gallus	DCLK1	SPSPT**SP**GSLR
xenopus_tropicalis	dclk1	CSSPT**SP**GSFR
petromyzon_marinus	DCLK1 (1 of 2)	SPAST**SP**GSLR
petromyzon_marinus	DCLK1 (2 of 2)	-----------
drosophila_melanogaster	CG17528	-----------
caenorhabditis_elegans	zyg-8	-----------

ENSRNOP00000029044[Mllt4]@622

rattus_norvegicus	**Mllt4**	HRPDI**SP**TERT
gallus_gallus	MLLT4	YRPDI**TP**TERT
pelodiscus_sinensis	MLLT4	YRPDI**SP**TERT
anolis_carolinensis	MLLT4	YRPDI**SP**AERT
xenopus_tropicalis	mllt4	YRPDT**SP**AERT
danio_rerio	MLLT4 (3 of 3)	-----------
danio_rerio	mllt4	YRPDI**SP**SERT
danio_rerio	MLLT4 (2 of 3)	SQRNK**S**HKQTE
petromyzon_marinus	mllt4	SLPSQQQQRRR
drosophila_melanogaster	Spn	SLPDDMTAAEA
drosophila_melanogaster	cno	YRPELQPTERA
caenorhabditis_elegans	afd-1	----------K
caenorhabditis_elegans	nab-1	-----------

ENSRNOP00000018697[Stmn3]@81

rattus_norvegicus	**Stmn3**	KRKDA**S**LEELQ
gallus_gallus	STMN3	KKKDL**S**LEELQ
pelodiscus_sinensis	STMN3	KKKDL**S**LEELQ
anolis_carolinensis	STMN3	KKRDL**S**LEELQ
xenopus_tropicalis	stmn3	TKKDI**S**LEELQ
danio_rerio	stmn3	QRKEP**S**LGELQ
drosophila_melanogaster	stai	--KNV**S**VEEIE

ENSRNOP00000031570[Mical3]@1188

rattus_norvegicus	**Mical3**	TTK--------------------VK**SP**AEEP
gallus_gallus	MICAL3	------------------------K**SP**-EAR
pelodiscus_sinensis	MICAL3	------------------------R**SP**-EKR
anolis_carolinensis	MICAL3	SCE--------------------ER**S**S-DTH
xenopus_tropicalis	mical3	-------------------------------
drosophila_melanogaster	Mical	TPRATLVSRNTEDRDYLDKLESLERD--EET

ENSRNOP00000035734[Clip2]@315

rattus_norvegicus	**Clip2**	SALTH**SP**SSSS
gallus_gallus	CLIP2	SALTH**SP**SSSS
pelodiscus_sinensis	CLIP2	SALTH**SP**SSSS
anolis_carolinensis	CLIP2	SALTH**SP**SSSS
danio_rerio	clip2	SSLAH**SP**SSSS
drosophila_melanogaster	CLIP-190	SRTGSRESLTS
drosophila_melanogaster	CG11242	-----------
caenorhabditis_elegans	F53F4.3	-----------

ENSRNOP00000026920[Hsp90ab1]@255

rattus_norvegicus	**Hsp90ab1**	IEDVG**S**DEEDD
gallus_gallus	HSP90AB1	IEDVG**S**DEEEE
pelodiscus_sinensis	HSP90AB1	IEDVG**S**DEEEE
anolis_carolinensis	HSP90AB1	IEDVG**S**DEEEE
xenopus_tropicalis	hsp90ab1	IEDVG**S**DEEEE
danio_rerio	hsp90ab1	IEDVG**S**DDEED
petromyzon_marinus	hsp90ab1	IEDVG**S**DEEED
drosophila_melanogaster	Hsp83	IEDVGEDEDAD
caenorhabditis_elegans	daf-21	VENVADDA---

ENSRNOP00000045121[Ctnnd2]@400

rattus_norvegicus	**Ctnnd2**	LRALQ**SP**EHHI
gallus_gallus	CTNND2	-----------
pelodiscus_sinensis	CTNND2	LRALQ**SP**EHHI
anolis_carolinensis	CTNND2	LRSLQ**SP**EHHI
xenopus_tropicalis	ctnnd2	IRSLQ**SP**EHHI
drosophila_melanogaster	p120ctn	-----------
caenorhabditis_elegans	jac-1	-----------

ENSRNOP00000044134[Robo1]@1240

rattus_norvegicus	**Robo1**	A-----ERGP**TP**---PVRG
gallus_gallus	ROBO1	ED----ERGP**TP**---PVRG
pelodiscus_sinensis	ROBO1	DD----ERGP**TP**---PVRG
anolis_carolinensis	ROBO1	ED----ERGP**TP**---PVRG
xenopus_tropicalis	robo1	------ERGP**TP**---PIRG
danio_rerio	robo1	DEVEL-ERGP**TP**---PVRG
petromyzon_marinus	robo1	DDEEADEEDDLV---VDEG
drosophila_melanogaster	robo	PMQ-------PP---PPVP
drosophila_melanogaster	lea	QNL-LDLDGS**S**M---CYNG
drosophila_melanogaster	Nrg	YSQPLDNKSAGR---QSVS
drosophila_melanogaster	robo3	NGQ-MDFLTGGP---PSEG
caenorhabditis_elegans	rig-6	-------------------
caenorhabditis_elegans	sax-7	YKTDDDEKRSLT-------
caenorhabditis_elegans	sax-3	------NRGS**TP**REDTYDS
caenorhabditis_elegans	lad-2	-------------------

ENSRNOP00000044341[Nrcam]@1136

rattus_norvegicus	**Nrcam**	TFGEY--------**S**DAEDH
gallus_gallus	NRCAM	TFGEYRSL----E**S**DAEDH
anolis_carolinensis	NRCAM	TLGEYRSL----E**S**DADDH
xenopus_tropicalis	nrcam	TFGEY--------**S**DAEDH
danio_rerio	NRCAM (3 of 3)	TFGEY--------------
drosophila_melanogaster	robo	NLTTFYNC----RKSPDNP
drosophila_melanogaster	lea	HIADYAPV-CGAPGSPAGG
drosophila_melanogaster	Nrg	GFHEY--------**S**QPLDN
drosophila_melanogaster	robo3	NMPDYAEVACSTFKSPTHG
caenorhabditis_elegans	rig-6	-------------------
caenorhabditis_elegans	sax-7	GKPDY--------KTDDDE
caenorhabditis_elegans	sax-3	AMSTFYG-----NQYHDDP
caenorhabditis_elegans	lad-2	QFLEY--------------

ENSRNOP00000009802[Cnksr2]@756

rattus_norvegicus	**CNKSR2**	GSSAV**SP**IRKT
gallus_gallus	CNKSR2	GSVTD**SP**VRKT
pelodiscus_sinensis	CNKSR2	GSTTE**SP**VRKT
anolis_carolinensis	CNKSR2	GSTTE**SP**IRKT
xenopus_tropicalis	cnksr2	GSTTD**SP**VRKT
drosophila_melanogaster	cnk	KPSSDIPV--P
caenorhabditis_elegans	cnk-1	GS---------

ENSRNOP00000020581[Dnajc5]@10

rattus_norvegicus	**Dnajc5**	R-------QR-SL**S**TSGES
gallus_gallus	DNAJC5	R-------QR-SL**S**TSGES
pelodiscus_sinensis	DNAJC5	R-------QR-SL**S**TSGES
anolis_carolinensis	DNAJC5	R-------QR-SL**S**TSGES
drosophila_melanogaster	CG7133	------------M**S**DVYED
drosophila_melanogaster	Csp	--SAPGMDKR-KL**S**TSGDS
drosophila_melanogaster	CG32640	---------------MEED
drosophila_melanogaster	CG32641	---------------MEED
caenorhabditis_elegans	dnj-16	ATMSKATTPGDQPKVSEMD
caenorhabditis_elegans	dnj-14	REESPAADHS-HDPKKGLH
caenorhabditis_elegans	dnj-24	------------MAPREDS

ENSRNOP00000022059[Grm5]@1016

rattus_norvegicus	**Grm5**	RPRSP**SP**ISTL
gallus_gallus	GRM5	RPQTP**SP**ISTV
anolis_carolinensis	GRM5	RAQTP**SP**ISTV
xenopus_tropicalis	grm5	RPQTP**SP**ISTV
danio_rerio	GRM5 (2 of 2)	RPQTP**SP**ISTV
danio_rerio	GRM5 (1 of 2)	RPRTP**SP**LPTI
drosophila_melanogaster	mtt	-----------
caenorhabditis_elegans	mgl-2	R----------

ENSRNOP00000011654[Nsfl1c]@114

rattus_norvegicus	**Nsfl1c**	PPRKK**SP**NELV
gallus_gallus	NSFL1C	PPRKK**SP**NELV
pelodiscus_sinensis	NSFL1C	PPRKK**SP**NELV
anolis_carolinensis	NSFL1C	PPRKK**SP**NELV
danio_rerio	nsfl1c	PPKKKNSNELI
danio_rerio	nsfl1c	PPKKKNSNELI
petromyzon_marinus	nsfl1c	PPRKRNPNEIV
drosophila_melanogaster	p47	PPKRKNFREQL
drosophila_melanogaster	CG42383	AGKRVNINSST
caenorhabditis_elegans	ubxn-2	P----------

ENSRNOP00000005519[Samd14]@140

rattus_norvegicus	**Samd14**	AAT-SG-**SP**P--RSA
gallus_gallus	SAMD14	---------------
anolis_carolinensis	SAMD14	GSTPTSS**SP**---KSC
danio_rerio	SAMD14	PLSPLK-**SP**S--RAC
drosophila_melanogaster	Spn	GNIRKE-AAN--LQN
drosophila_melanogaster	cno	MNAYNGS**SP**LAPQQQ
caenorhabditis_elegans	nab-1	DVTHKK-AEQ--YHE
caenorhabditis_elegans	afd-1	GSSGRN-**S**AFVAPLS

ENSRNOP00000050980[Myo18a]@2010

rattus_norvegicus	**MYO18A**	LAPDL**S**DDEHD
gallus_gallus	MYO18A	FSYDRWDEELD
pelodiscus_sinensis	MYO18A	-----------
anolis_carolinensis	MYO18A	-----------
xenopus_tropicalis	MYO18A	QSRTPVSDEAG
drosophila_melanogaster	Mhcl	IPANKPDTKPA

ENSRNOP00000002895[Ncam2]@747

rattus_norvegicus	**Ncam2**	YL---KDG**S**KEPI-V
gallus_gallus	NCAM2	YL---KDG**S**KEPI-V
pelodiscus_sinensis	NCAM2	YL---KDG**S**KEPI-V
anolis_carolinensis	NCAM2	---
xenopus_tropicalis	ncam2	YLW--KDG**S**KEPI-V
danio_rerio	ncam2	YL---SQN**T**KEPIDC
danio_rerio	NCAM2 (2 of 2)	YL---VDG**S**KEPI-V
drosophila_melanogaster	CG8964	---
drosophila_melanogaster	Fas2	LV---KEPPPSPL--
drosophila_melanogaster	CG33543	---
drosophila_melanogaster	zormin	GIRPGFKRAPSEGSSQQPQAFRDESRVSQYGTKCVDPNTGLIYFKYDFGYEFGILFPGEGHKFVSSQWNSSGSSNSNAKPIQNGRHSPYPL
drosophila_melanogaster	CG15630	---
drosophila_melanogaster	otk	LL---
caenorhabditis_elegans	ncam-1	---

ENSRNOP00000002895[Ncam2]@768

rattus_norvegicus	**Ncam2**	NHEDG**S**-**P**V-NEP
gallus_gallus	NCAM2	SHEDG**S**-**P**V-NEP
pelodiscus_sinensis	NCAM2	NHEDG**S**-**P**V-NEP
anolis_carolinensis	NCAM2	-------------
xenopus_tropicalis	ncam2	NH-DG**S**-**P**V-NEP
danio_rerio	ncam2	SAEYCPIPI-NSV
danio_rerio	NCAM2 (2 of 2)	NPEDC**S**-**P**V-NEP
drosophila_melanogaster	CG8964	-------PCLTEA
drosophila_melanogaster	Fas2	-------PL-PPP
drosophila_melanogaster	CG33543	-------------
drosophila_melanogaster	zormin	-------PVQHER
drosophila_melanogaster	CG15630	-------PF----
drosophila_melanogaster	otk	-------PI----
caenorhabditis_elegans	ncam-1	-------P---ES

ENSRNOP00000009587[Tmx1]@245

rattus_norvegicus	**Tmx1**	DEEDV**S**EEETE
gallus_gallus	TMX1	-----------
anolis_carolinensis	TMX1	PPQTA**S**EEFSE
xenopus_tropicalis	tmx1	KEEVNEFENTK
danio_rerio	tmx1	-----------
petromyzon_marinus	tmx1	-----------
drosophila_melanogaster	CG5554	LEDLA**T**EEIED
caenorhabditis_elegans	dpy-11	GTESP**T**KKNGN

ENSRNOP00000040859[Canx]@563

rattus_norvegicus	**Canx**	DGGTG**S**QD-----------EED
gallus_gallus	CANX	DIGSA**S**QEEEEEEEE----EED
pelodiscus_sinensis	CANX	DVGSA**S**QEEEEEEEE----EED
anolis_carolinensis	CANX	EAETA**S**QDEEEEEEEEEGGGEEE
xenopus_tropicalis	canx	-----AED-----------QES
danio_rerio	canx	-------------------DDN
drosophila_melanogaster	Cnx14D	-QKSYLDNNEDEEDT----LKN
drosophila_melanogaster	Crc	-------------------ESK
drosophila_melanogaster	Cnx99A	-QKSDLDDNEEESKA----AES
drosophila_melanogaster	CG1924	PPKKKLDDNEWKFKA----AKK
caenorhabditis_elegans	cnx-1	SQSSAAEEEDDEH--V---VPE
caenorhabditis_elegans	crt-1	-------------------EEG

ENSRNOP00000065381[Dcx]@332

rattus_norvegicus	**Dcx**	PKSKQ**SP**ISTP
gallus_gallus	DCX	PKSKQ**SP**ISTP
pelodiscus_sinensis	DCX	PKSKQ**SP**ISTP
anolis_carolinensis	DCX	PKSKQ**SP**ISTP
xenopus_tropicalis	dcx	PKSKQ**SP**ISTP
drosophila_melanogaster	CG17528	PLVDSEPFKAE
caenorhabditis_elegans	zyg-8	--------SGS

ENSRNOP00000061003[Robo2]@1044

rattus_norvegicus	**Robo2**	EDRVP-**TP**PVRG
gallus_gallus	ROBO2	DDRVP-**TP**PVRG
pelodiscus_sinensis	ROBO2	DDRVP-**TP**PVRG
anolis_carolinensis	ROBO2	DDRVP-**TP**PVRG
xenopus_tropicalis	ROBO2	EERVP-**TP**PVRG
danio_rerio	robo2	------------
danio_rerio	robo2	EERVP-**TP**PIRG
petromyzon_marinus	robo2	------------
drosophila_melanogaster	robo	FSAWGV**SP**-QYA
drosophila_melanogaster	lea	KQSSPI**S**S-QFA
drosophila_melanogaster	Nrg	------------
drosophila_melanogaster	robo3	YANTTLAA-QMA
caenorhabditis_elegans	rig-6	------------
caenorhabditis_elegans	sax-7	------------
caenorhabditis_elegans	sax-3	QRS---------
caenorhabditis_elegans	lad-2	------------

ENSRNOP00000061003[Robo2]@1180

rattus_norvegicus	**Robo2**	RQRPT**SP**FSTD
gallus_gallus	ROBO2	RPRPS**SP**FSTD
pelodiscus_sinensis	ROBO2	RPRPS**SP**FSTD
anolis_carolinensis	ROBO2	RPRPT**SP**FSTD
xenopus_tropicalis	ROBO2	RPRPA**SP**FSTD
danio_rerio	robo2	-----------
danio_rerio	robo2	RPRPA**SP**FSTD
petromyzon_marinus	robo2	-----------
drosophila_melanogaster	robo	PPPVPVPEGWY
drosophila_melanogaster	lea	MCYNGLADSGC
drosophila_melanogaster	Nrg	----A**T**AAARA
drosophila_melanogaster	robo3	PPSEGGDFSGL
caenorhabditis_elegans	rig-6	-----------
caenorhabditis_elegans	sax-7	----F**T**EDGSF
caenorhabditis_elegans	sax-3	DTYDSVSDGAF
caenorhabditis_elegans	lad-2	-----------

ENSRNOP00000012131[Ehbp1]@335

rattus_norvegicus	Ehbp1	VMI-KD**SP**PQST
gallus_gallus	EHBP1	VTV-RD**SP**PQPA
pelodiscus_sinensis	EHBP1	VTI-RD**SP**PQPA
anolis_carolinensis	EHBP1	ASM-KDFPPQPA
xenopus_tropicalis	ehbp1	LELSKD**SP**PQPA
danio_rerio	ehbp1	P---KV**SP**PRSS
petromyzon_marinus	ehbp1	------------
drosophila_melanogaster	Ehbp1	VESVKE------
caenorhabditis_elegans	ehbp-1	VSAEEEKTSQPA

ENSRNOP00000017102[Amph]@239

rattus_norvegicus	**Amph**	IAKTP----**SP**PEEA
gallus_gallus	AMPH	IAKTP----**SP**PEEV
pelodiscus_sinensis	AMPH	IAKTP----**SP**PEEA
anolis_carolinensis	AMPH	IAKTP----**SP**PDEE
xenopus_tropicalis	amph	IAKTP----**SP**PEEV
danio_rerio	amph	LARTP----**SP**PDDE
petromyzon_marinus	amph (2 of 2)	ITETP---C**SP**PEIT
petromyzon_marinus	amph (1 of 2)	TREPPPSIVEPPAGT
drosophila_melanogaster	Amph	---------------
caenorhabditis_elegans	amph-1	VARPEA--R------

ENSRNOP00000025878[Ap3d1]@825

rattus_norvegicus	**Ap3d1**	A---ETVK**SP**-----------EKEG
gallus_gallus	AP3D1	A---EALKMP-----------ESE-
pelodiscus_sinensis	AP3D1	A---ENLK**SP**-----------DTE-
anolis_carolinensis	AP3D1	A---EGLK**SP**----------QEAD-
xenopus_tropicalis	ap3d1	A---EILK**SP**-----------ENE-
danio_rerio	ap3d1	P---EVLK**SP**-------AEDRDDNT
petromyzon_marinus	ap3d1 (1 of 2)	V---AKSTGP-----------ESLY
petromyzon_marinus	ap3d1 (2 of 2)	-------------------------
drosophila_melanogaster	g	AADKENLK**TP**-----------ADSA
caenorhabditis_elegans	apd-3	P---LVPRPPTTFEPRLVAQQQEPA

ENSRNOP00000011573[Gabbr2]@883

rattus_norvegicus	**Gabbr2**	IED----------------IN**SP**EHIQ
gallus_gallus	GABBR2	IED----------------IN**SP**EHIQ
pelodiscus_sinensis	GABBR2	IED----------------IN**SP**EHIQ
anolis_carolinensis	GABBR2	IED----------------IN**SP**EHIQ
xenopus_tropicalis	gabbr2	IED----------------IN**SP**EHIQ
danio_rerio	gabbr2	LED----------------IN**SP**EHIQ
petromyzon_marinus	gabbr2 (1 of 2)	---------------------------
petromyzon_marinus	gabbr2 (2 of 2)	---------------------------
drosophila_melanogaster	GABA-B-R2	IQQQLQQHLQQHQQMQQQHLQQQQHQQ
drosophila_melanogaster	GABA-B-R1	ASL----------------IN**S**SAH--
drosophila_melanogaster	GABA-B-R3	MRRSVT--FASQPQLEEACLPAQDLIN
caenorhabditis_elegans	gbb-2	NGE------------------------
caenorhabditis_elegans	gbb-1	LID----------------LQNGNH--

ENSRNOP00000060014[Slc9a6]@700

rattus_norvegicus	**Slc9a6**	DDTRH**SP**A
gallus_gallus	SLC9A6	DSARHGPA
pelodiscus_sinensis	SLC9A6	DSARHGPA
anolis_carolinensis	SLC9A6	DNARHGPA
xenopus_tropicalis	slc9a6	VTRRQ**SP**K
drosophila_melanogaster	Nhe1	--------
drosophila_melanogaster	Nhe3	----NNFM
caenorhabditis_elegans	nhx-5	--------
caenorhabditis_elegans	nhx-8	SDSSTNEF
caenorhabditis_elegans	nhx-4	DESE----
caenorhabditis_elegans	pbo-4	TESEE**T**SF

ENSRNOP00000060347[Neo1]@1155

rattus_norvegicus	**Neo1**	KPIDK**SP**DPNP
gallus_gallus	NEO1	KPIDK**SP**DPNP
pelodiscus_sinensis	NEO1	KPIDK**SP**DPNP
anolis_carolinensis	NEO1	KPIDK**SP**DPNP
xenopus_tropicalis	neo1	KPTDK**SP**ETNP
danio_rerio	neo1	KPIDK**SP**EPNP
danio_rerio	NEO1 (2 of 2)	KPMDK**SP**EPNP
petromyzon_marinus	neo1	-----------
drosophila_melanogaster	fra	KNIDKG-----
drosophila_melanogaster	Cont	-----------
caenorhabditis_elegans	unc-40	RAGASDYMVDG

ENSRNOP00000055734[Stmn4]@117

rattus_norvegicus	**Stmn4**	RR-----RDP**S**LEEIQ
gallus_gallus	STMN4	RR-----RDP**S**LEEIQ
pelodiscus_sinensis	STMN4	RR-----RDP**S**LEEIQ
xenopus_tropicalis	stmn4	QK-----RDP**S**LEEIQ
danio_rerio	stmn4	QR-----KDP**S**LEEIQ
petromyzon_marinus	stmn4	RK-----KDL**S**LEEIL
drosophila_melanogaster	stai	KRPVTPGKNV**S**VEEIE

ENSRNOP00000063519[Ctnnd1]@47

rattus_norvegicus	**Ctnnd1**	ERV--RV**SP**QDAN
gallus_gallus	CTNND1	---
pelodiscus_sinensis	CTNND1	ERV--RV**SP**QDAS
anolis_carolinensis	CTNND1	ERV--RV**SP**QDAS
xenopus_tropicalis	ctnnd1	DRV--WV**TP**QEPP
danio_rerio	ctnnd1	---
danio_rerio	ctnnd1	---
drosophila_melanogaster	p120ctn	---
caenorhabditis_elegans	jac-1	QKVTRVTKVTTTRSVRQVPVQSPYSNIDFDSSGLPTPSPVIDRDPSLEMMARMGGASDHDSEDRSAPPPAP

ENSRNOP00000012644[Hcn4]@1024

rattus_norvegicus	**Hcn4**	PRGGL**SP**PGHS
gallus_gallus	HCN4	P-SGL**SP**PTQS
pelodiscus_sinensis	HCN4	P-SGL**SP**PSQS
anolis_carolinensis	HCN4	P-SGL**SP**PHHS
xenopus_tropicalis	hcn4	-----------
danio_rerio	hcn4	P-LAL**SP**TVQS
petromyzon_marinus	hcn4	-----------
drosophila_melanogaster	Ih	-----------

ENSRNOP00000062549[Tjp2]@107

rattus_norvegicus	**Tjp2**	APL-QG**SP**-----------------PLSH
gallus_gallus	TJP2	AAL-RR**SP**-----------------SLDY
pelodiscus_sinensis	TJP2	TVL-RR**SP**-----------------SLDY
anolis_carolinensis	TJP2	PAV-QR**SP**-----------------SLDY
xenopus_tropicalis	tjp2	TA--RR**SP**-----------------SPEL
drosophila_melanogaster	CG6509	KTRDRDCPHGLQLDMGVFIAKIEQNSLAF
drosophila_melanogaster	pyd	----RRVP---------------------
caenorhabditis_elegans	zoo-1	----VAVP-----------------MLEY

ENSRNOP00000066019[Ccdc28b]@46

rattus_norvegicus	**Ccdc28b**	LPHLL**SP**KQRA
gallus_gallus	CCDC28B	LPALP**S**--QHA
pelodiscus_sinensis	CCDC28B	LPHLP**SP**KQRA
anolis_carolinensis	CCDC28B	RPHLP**SP**KQRA
xenopus_tropicalis	ccdc28b	LPHPP**SP**KQRG
danio_rerio	ccdc28b	LPQPP**SP**KPRG
petromyzon_marinus	ccdc28b	-----------
drosophila_melanogaster	CG10874	SSEWLAPDPTA

ENSRNOP00000026016[Ctnnb1]@552

rattus_norvegicus	**Ctnnb1**	TQRR-----T**S**MGGTQ
gallus_gallus	CTNNB1	TQRR-----T**S**MGGTQ
pelodiscus_sinensis	CTNNB1	TQRR-----T**S**MGGTQ
anolis_carolinensis	CTNNB1	TQRR-----T**S**MGGTQ
xenopus_tropicalis	ctnnb1	TQRR-----T**S**MGGTQ
danio_rerio	ctnnb1	TQRR-----T**S**MGGTQ
petromyzon_marinus	CTNNB1 (1 of 5)	TQRQ-----GVAANGQ
petromyzon_marinus	CTNNB1 (2 of 5)	TQRH-----TMGTMQQ
petromyzon_marinus	CTNNB1 (5 of 5)	----------------
petromyzon_marinus	CTNNB1 (4 of 5)	TLRT-------ASGTL
petromyzon_marinus	CTNNB1 (3 of 5)	TQRQ-----SAGSGGQ
drosophila_melanogaster	arm	TERQRSSIAT**T**GSQQP
caenorhabditis_elegans	hmp-2	IEEN-----------P
caenorhabditis_elegans	bar-1	LNVQ------------

ENSRNOP00000017101[Pgrmc1]@181

rattus_norvegicus	**Pgrmc1**	EPTVY**S**DDEEP
gallus_gallus	PGRMC1	EPTVY**S**DEEEK
pelodiscus_sinensis	PGRMC1	EPTVY**S**DEEEE
anolis_carolinensis	PGRMC1	EPTVY**S**DGEET
xenopus_tropicalis	pgrmc1	-----------
danio_rerio	pgrmc1	EPTEY**T**DDEEV
drosophila_melanogaster	MSBP	EPTNYDDDEDE
drosophila_melanogaster	CG16957	EKLQINEEEDF
caenorhabditis_elegans	vem-1	EKADYGNRKSF

ENSRNOP00000024850[Trim3]@427

rattus_norvegicus	**Trim3**	GDLPP**SP**DD-VK
gallus_gallus	TRIM3	SDVPP**SP**DD-VK
anolis_carolinensis	TRIM3	SDVPP**SP**DD-VK
xenopus_tropicalis	trim3	GDVPP**SP**ED-VK
drosophila_melanogaster	abba	NELAS**S**DDDGSK
drosophila_melanogaster	brat	---VSGAGA---
drosophila_melanogaster	CG8419	HT----------
drosophila_melanogaster	wech	------------
caenorhabditis_elegans	nhl-1	------------
caenorhabditis_elegans	nhl-3	YDW---------
caenorhabditis_elegans	lin-41	YD----------
caenorhabditis_elegans	ncl-1	---SVGGLL---

ENSRNOP00000007002[Slc1a4]@499

rattus_norvegicus	**Slc1a4**	IPN---------------------SK**S**EEETS
gallus_gallus	SLC1A4	VAN---------------------SK**S**EEETS
pelodiscus_sinensis	SLC1A4	IPN---------------------CK**S**EEETS
anolis_carolinensis	SLC1A4	VPN---------------------IKAEGETS
xenopus_tropicalis	slc1a4	VPN---------------------LK**S**EEETS
danio_rerio	slc1a4	VAN---------------------VKAEEETS
drosophila_melanogaster	Eaat2	MPAGTPNGHGHGHGHHDGGLLEGQTELETSSK
drosophila_melanogaster	Eaat1	LLELGPNGH-----------------------
caenorhabditis_elegans	glt-3	IRE-------------DIDIL---NNPHQDTL
caenorhabditis_elegans	glt-4	------------------------TNDQGYSM
caenorhabditis_elegans	glt-1	--------------------------AATTRS
caenorhabditis_elegans	glt-5	------------------------KNHAVVEA
caenorhabditis_elegans	glt-6	LKG-------------EIELL---KSAATSRR
caenorhabditis_elegans	glt-7	----------------EGDL------AEIDRP

ENSRNOP00000017199[Rras2]@186

rattus_norvegicus	**Rras2**	QEC-----PP**SP**EPTR
gallus_gallus	RRAS2	QEC-----PP**SP**EPTR
pelodiscus_sinensis	RRAS2	QEC-----PP**SP**EPTR
anolis_carolinensis	RRAS2	QEC-----PP**SP**EPTR
xenopus_tropicalis	RRAS2	QEC-----PP**SP**EPTR
danio_rerio	rras2	QEC-----PP**SP**EPTR
petromyzon_marinus	rras2	DEC-----PP**S**SEPPT
drosophila_melanogaster	Ras85D	NKG---------RRGR
drosophila_melanogaster	Ras64B	AER-----PFIEQDYK
caenorhabditis_elegans	let-60	------------RERH
caenorhabditis_elegans	ras-2	DEGDHEASMA**S**VPRTK
caenorhabditis_elegans	ras-1	DER----MPIHPHDDR

ENSRNOP00000025209[Slc6a7]@600

rattus_norvegicus	**Slc6a7**	LAGSQ**SP**K-PLM
gallus_gallus	SLC6A7	LAGSQ**SP**K-PLM
pelodiscus_sinensis	SLC6A7	LAGSQ**SP**K-PLM
anolis_carolinensis	SLC6A7	LAGSQ**SP**K-PLM
xenopus_tropicalis	slc6a7	LAGSQ**SP**K-PLM
danio_rerio	SLC6A7	SSQEQHVE-PHT
drosophila_melanogaster	SerT	------------
drosophila_melanogaster	DAT	LTDTE**T**AKEPVD
caenorhabditis_elegans	dat-1	---------PHS
caenorhabditis_elegans	snf-3	AEELKFIE----
caenorhabditis_elegans	mod-5	LLDPIHTLTPV-

ENSRNOP00000036787[Mfsd6]@11

rattus_norvegicus	**Mfsd6**	KVAIL**T**D-----------------------------------DE--------EE
gallus_gallus	MFSD6	KVAIL**T**D-----------------------------------DE--------EE
pelodiscus_sinensis	MFSD6	KVAIL**T**D-----------------------------------DE--------EE
anolis_carolinensis	MFSD6	KVAIL**T**D-----------------------------------DE--------ED
xenopus_tropicalis	mfsd6	KVAIL**S**E-----------------------------------DE--------ED
drosophila_melanogaster	CG31663	MVS---EE
drosophila_melanogaster	CG12858	YTAGA**S**GGVGGGGGANPFGAPAGGAGYGQQGYGYEQQATGGYDQGMNYGIEPQQ
drosophila_melanogaster	Sug	--
drosophila_melanogaster	CG15706	--
caenorhabditis_elegans	R13A5.9	HVG---ER

ENSRNOP00000028785[Rtn3]@31

rattus_norvegicus	Rtn3	GG---GGG**SP**GACP
pelodiscus_sinensis	RTN3	--
anolis_carolinensis	RTN3	GGR---DLKGSG**S**HQSCA
xenopus_tropicalis	rtn3	--
danio_rerio	rtn3	--
danio_rerio	rtn3	--
petromyzon_marinus	RTN3 (2 of 2)	--
petromyzon_marinus	RTN3 (1 of 2)	--
drosophila_melanogaster	CG42853	--
drosophila_melanogaster	Rtnl1	GG---DTGNVSG--
drosophila_melanogaster	Rtnl2	--
caenorhabditis_elegans	ret-1	EAPAVFGLEDSEDIINKHALETASNQEFSKIKEPEVTEVSENTAENLNEEVGESKNPEAIDLVESGENAGIE

ENSRNOP00000042012[Slc8a2]@622

rattus_norvegicus	Slc8a2	LKRGISALLL---N
pelodiscus_sinensis	SLC8A2	LKRGISALLL---T
anolis_carolinensis	SLC8A2	LKRGISALLL---N
xenopus_tropicalis	slc8a2	MKRGISAALL----------------LNQECWWNISLALGKHIYTKVAIHKLSLRLSVFPGNLSAITAIT
drosophila_melanogaster	Calx	APDDTNKESF---FNRFFEK-------HEHSGEPTHDELAA----------------------------K
caenorhabditis_elegans	ncx-1	--
caenorhabditis_elegans	ncx-3	--
caenorhabditis_elegans	ncx-2	AKKMNDLSRIQERFQRRMERKRGSSVASESKDSNTENALAPAEKSTRAASVD----------------LL

ENSRNOP00000003667[Pbdc1]@184

rattus_norvegicus	**Pbdc1**	EKGAD**S**GGEKE
gallus_gallus	PBDC1	-----------
pelodiscus_sinensis	PBDC1	ETGGSA-----
danio_rerio	pbdc1	DKS--------
drosophila_melanogaster	CG7519	EEQ--------
caenorhabditis_elegans	Y54E5A.5	SL---------

ENSRNOP00000019077[Ahi1]@182

rattus_norvegicus	**Ahi1**	ITGRD**SP**VHPK
gallus_gallus	AHI1	MRTSEISAQSK
pelodiscus_sinensis	AHI1	MNSPEVYIQSK
anolis_carolinensis	AHI1	VASSV**SP**-EPK
xenopus_tropicalis	ahi1	E------KHPK
danio_rerio	ahi1	-------TEEK
petromyzon_marinus	ahi1	RRE--------
drosophila_melanogaster	CG34133	-----------
caenorhabditis_elegans	sym-4	----------K

ENSRNOP00000018796[Pgrmc2]@205

rattus_norvegicus	**Pgrmc2**	EPSEY**T**DEEDT
gallus_gallus	PGRMC2	EPSEY**T**DEEDT
pelodiscus_sinensis	PGRMC2	EPSEY**T**DEEDI
anolis_carolinensis	PGRMC2	EPSEY**T**DEEDT
xenopus_tropicalis	pgrmc2	EPSEY**T**DEEDV
danio_rerio	pgrmc2	EPSEY**T**DEEDM
drosophila_melanogaster	MSBP	EPTNYDDDEDE
drosophila_melanogaster	CG16957	EKLQINEEEDF
caenorhabditis_elegans	vem-1	EKADYGNRKSF

ENSRNOP00000063572[Atp2b2]@1257

rattus_norvegicus	**Atp2b2**	LKQNS**SP**PSSL
gallus_gallus	ATP2B2	-----------
anolis_carolinensis	ATP2B2	-----------
xenopus_tropicalis	atp2b2	-KGLSKAHKDQ
danio_rerio	atp2b2	-RKNS**S**QPASP
petromyzon_marinus	atp2b2	-----------
drosophila_melanogaster	PMCA	-TLHNGLTSQR
caenorhabditis_elegans	mca-3	-TAAAAAPKAE
caenorhabditis_elegans	mca-2	-GLRA**S**GQVYS
caenorhabditis_elegans	mca-1	-------EKKK

ENSRNOP00000060362[Trim2]@445

rattus_norvegicus	**Trim2**	RS-----------------------------ADV**SP**TTEG
gallus_gallus	TRIM2	RS-----------------------------ADV**SP**TTEG
pelodiscus_sinensis	TRIM2	RS-----------------------------ADV**SP**TTEG
anolis_carolinensis	TRIM2	RS-----------------------------ADV**SP**TTEG
xenopus_tropicalis	trim2	KS-----------------------------ADV**SP**TTEG
drosophila_melanogaster	abba	ESEDRYGSGTGSSYTSRFLNKSKSSAIVTQPSLS**TP**TASF
drosophila_melanogaster	brat	-------------------------------VGG**S**VVSGA
drosophila_melanogaster	CG8419	-------------------------------IKGGPFTFQ
drosophila_melanogaster	wech	PRT----------------------------TQAIPGCMD
caenorhabditis_elegans	nhl-1	PASAPITNGTSE-------------------QVAIPVTHF
caenorhabditis_elegans	nhl-3	-------------------------------VAN**SP**LAFR
caenorhabditis_elegans	lin-41	-------------------------------ISGCPTTMD
caenorhabditis_elegans	ncl-1	KWS----------------------------LGVEPSVGG

ENSRNOP00000000874[Svop]@25

rattus_norvegicus	**Svop**	GESAR**S**EDDAA
gallus_gallus	SVOP	GESSR**S**EEDAI
anolis_carolinensis	SVOP	-----------
xenopus_tropicalis	svop	GESSK**S**EDDNI
drosophila_melanogaster	CG15221	-----------
drosophila_melanogaster	CG12783	-----------
drosophila_melanogaster	CG4324	-----------
drosophila_melanogaster	CG33234	-----------
drosophila_melanogaster	CG33233	-----------
drosophila_melanogaster	CG14691	-----------
drosophila_melanogaster	CG31272	-----------
drosophila_melanogaster	CG3168	---AT**S**HIDNR
drosophila_melanogaster	CG3690	-----------
drosophila_melanogaster	CG31103	-----------
drosophila_melanogaster	CG31106	-----------
caenorhabditis_elegans	svop-1	---TEAYVDLT

ENSRNOP00000019225[Kif2a]@139

rattus_norvegicus	**Kif2a**	SVSDI**SP**---------------------VQAA
gallus_gallus	KIF2A	TVSDI**SP**---------------------VQAA
pelodiscus_sinensis	KIF2A	SVSDI**SP**---------------------VQAA
anolis_carolinensis	KIF2A	SVSDI**SP**---------------------VQAA
xenopus_tropicalis	kif2a	SVSDI**SP**---------------------DQPG
danio_rerio	KIF2A	TQSQL**T**Q---------------------QQQQ
drosophila_melanogaster	Klp10A	RIASAVPNNTLPNPSAAASAGPAAQGVATAAT
drosophila_melanogaster	Klp59D	--RDA**SP**---------------------TQGR
drosophila_melanogaster	Klp59C	VLATAPPRQQT----------------------
caenorhabditis_elegans	klp-7	TTAAFKP---------------------DLDS

ENSRNOP00000026960[Sgta]@81

rattus_norvegicus	**Sgta**	RGPDR**TP**------PSE----------------------E
gallus_gallus	SGTA	ANSEPVT------PSE----------------------D
pelodiscus_sinensis	SGTA	TNSEPVT------PSE----------------------E
anolis_carolinensis	SGTA	PTPEPVT------PSE----------------------E
xenopus_tropicalis	sgta	--SGLAS------PCD----------------------E
danio_rerio	sgta	TFVTTGS------PYE----------------------H
danio_rerio	SGTA (1 of 2)	---------------------------------------
drosophila_melanogaster	CG34274	GKAKRAL-------INTNQFTFMRQIDSEPDDRVLAREQ
drosophila_melanogaster	CG34297	EKSKPRTFNRI--DRSKDRFPFMRQVEMDLDQRSKARLE
drosophila_melanogaster	spag	SSSAA**S**S------PTE------KQDLPVDPV------AQ
drosophila_melanogaster	CG31294	KKSSRYLK-RVQKMSNINQISFMRQIDVSPKDRAEARRD
drosophila_melanogaster	CG6980	KQVK-----------DMNQKSFMEQVEKDANDRAEARAK
drosophila_melanogaster	Sgt	GAAAAAV------PNNIDMFELFQSLYTERN------PE
caenorhabditis_elegans	sgt-1	GESALPT------PSD----------------------S

ENSRNOP00000062744[Myh10]@1956

rattus_norvegicus	**Myh10**	ASLEL**S**DDDTE
gallus_gallus	MYH10	ASLEL**S**DDDAE
pelodiscus_sinensis	MYH10	ASLEL**S**DDDAE
anolis_carolinensis	MYH10	ASLEL**S**DDDAE
xenopus_tropicalis	myh10	ASLDI**S**DDEIE
danio_rerio	myh10	---DF**S**DDDAD
petromyzon_marinus	myh10	-----------
drosophila_melanogaster	zip	GGGGGDDSSVQ
drosophila_melanogaster	Mhc	LAFPPR---F-
caenorhabditis_elegans	myo-1	-----------
caenorhabditis_elegans	myo-6	-----------
caenorhabditis_elegans	myo-5	GGSRGA---FL
caenorhabditis_elegans	myo-2	-----------
caenorhabditis_elegans	myo-3	SSSNAR---FL
caenorhabditis_elegans	hum-9	-----------
caenorhabditis_elegans	nmy-2	SNTSLARDEFR
caenorhabditis_elegans	nmy-1	SSDNLAREEEN
caenorhabditis_elegans	unc-54	SPSRARASDF-

ENSRNOP00000047788[Gabbr1]@960

rattus_norvegicus	**Gabbr1**	RRH-----------PP**TP**P-----------------------DP--S
anolis_carolinensis	GABBR1	RRR-----------SSNCA----------------ADNHFNNSA--S
xenopus_tropicalis	gabbr1	RRR-----------PSVPA----------------RDN---
petromyzon_marinus	GABBR1 (1 of 2)	---
petromyzon_marinus	GABBR1 (2 of 2)	---
drosophila_melanogaster	GABA-B-R2	SRHYDS-------GSQ**TP**T-----------ARPKYSSSHRNSSTNISTSQSELSNMCPHSKP-STPAVIKTPTASDHRRTSMGSALKSNFVVSQSDLWDTHTLSHAKQRQSPRNYASPQRCAEHHGG
drosophila_melanogaster	GABA-B-R1	-------------------------------------TELNGAT--G
drosophila_melanogaster	GABA-B-R3	FKSHMGLFTRLIPSSQ**T**ASCNAIYNNPNQDSIPSEASSHPNGNHL---------------KPIHRGSLTKSGTHLDH-LTKDPNFLPIPTISGGEQGDQTLGGKYVKLLE----------------T
caenorhabditis_elegans	gbb-2	LRQ--------------------------------PSSSNNREE--T
caenorhabditis_elegans	gbb-1	-------------------------------------TEDMNAQL----------LCENDKQIADENL--T

ENSRNOP00000003189[Slc15a2]@28

rattus_norvegicus	**Slc15a2**	LPRPP**SP**PKKS
gallus_gallus	SLC15A2	PPKGDFHVQKK
pelodiscus_sinensis	SLC15A2	-----------
anolis_carolinensis	SLC15A2	PPKGICPPMKK
xenopus_tropicalis	slc15a2	PGRNPQYGSQN
danio_rerio	slc15a2	VDAEKYEKAQR
drosophila_melanogaster	yin	GQNGKNGQKEE
drosophila_melanogaster	CG2930	PDQNDKEPDQN
drosophila_melanogaster	CG9444	AQLKLAHRSSL
caenorhabditis_elegans	pept-1	ESVSSKGKTSY
caenorhabditis_elegans	pept-3	-----------
caenorhabditis_elegans	pept-2	---------SH

ENSRNOP00000020785[Usp5]@623

rattus_norvegicus	**Usp5**	A-P--PLV**TP**D-EPK
gallus_gallus	USP5	A-P--PLV**TP**D-EPK
pelodiscus_sinensis	USP5	A-P--PLV**TP**D-EPK
anolis_carolinensis	USP5	A-P--PLV**TP**D-EPK
xenopus_tropicalis	usp5	A-P--PLV**TP**D-EPK
danio_rerio	usp5	APP--PLM**TP**DVEVK
drosophila_melanogaster	CG12082	A-------**T**EE----
caenorhabditis_elegans	usp-5	E-PTAPRT**TP**D----

ENSRNOP00000043053[Phactr1]@237

rattus_norvegicus	**Phactr1**	LPVKL**SP**--PLPP
gallus_gallus	PHACTR1	MPVKL**SP**--PLPP
xenopus_tropicalis	phactr1	MPGKM**SP**--PLPP
drosophila_melanogaster	CG32264	PPIPL**S**EIGPIPP
caenorhabditis_elegans	F26H9.2	-PAIF**T**A--PPPP

ENSRNOP00000024261[Sept2]@218

rattus_norvegicus	**Sept2**	LPDAE**S**DEDED
gallus_gallus	SEPT2	LPDAE**S**DEDED
gallus_gallus	SEPT2	LPDAD**S**DEDEE
pelodiscus_sinensis	SEPT2	LPDAE**S**DEDED
anolis_carolinensis	SEPT2	LPDAE**S**DEDED
xenopus_tropicalis	sept2	LPDAE**S**DEDED
danio_rerio	sept2	LPDAE**S**DEDED
drosophila_melanogaster	Sep4	FPECD**S**DEDDD
drosophila_melanogaster	Sep1	LPDCD**S**DEDED
drosophila_melanogaster	pnut	FPATLEDAAEE
caenorhabditis_elegans	unc-59	FPELEDPYTD-

ENSRNOP00000018146[Plcl2]@458

rattus_norvegicus	**Plcl2**	VEGDV**T**DEDEG
gallus_gallus	PLCL2	LEGDV**T**DEDEG
pelodiscus_sinensis	PLCL2	LEGDV**T**XXXXX
anolis_carolinensis	PLCL2	LEGDV**T**DEDEG
xenopus_tropicalis	plcl2	IEGDV**T**DEDEG
danio_rerio	PLCL2	PEGEV**T**DEDEG
caenorhabditis_elegans	plc-4	EDIEEPDEDDS
caenorhabditis_elegans	pll-1	DSGEV**S**EEDDS

ENSRNOP00000040751[Slc8a1]@282

rattus_norvegicus	**Slc8a1**	GDRPA**S**KTE--IE
pelodiscus_sinensis	SLC8A1	GDRPS**S**KAD--IE
anolis_carolinensis	SLC8A1	GEGPQ**S**KAD--IE
xenopus_tropicalis	slc8a1	GDRPS**S**KAD--IE
drosophila_melanogaster	Calx	HDQ--------VE
caenorhabditis_elegans	ncx-1	RRSPSKKTRENVE
caenorhabditis_elegans	ncx-3	DDQPEKLSD----
caenorhabditis_elegans	ncx-2	AEE--------MK

ENSRNOP00000026063[Stx1b]@14

rattus_norvegicus	**Stx1b**	R--SAKD**S**DDEEE
pelodiscus_sinensis	STX1B	RRGSAKD**S**DDEEE
anolis_carolinensis	STX1B	R--SAKD**S**DDEEE
xenopus_tropicalis	stx1b	R--EAKD**S**DDDEE
danio_rerio	stx1b	R--SAKD**S**DDDEE
petromyzon_marinus	stx1b	-------------
drosophila_melanogaster	Syx1A	H--AAQ-**S**DDEEE
caenorhabditis_elegans	unc-64	K--AAQ-**S**EDEQD

ENSRNOP00000016417[Prkcb]@573

rattus_norvegicus	**Prkcb**	HPPVL**TP**P-DQE
gallus_gallus	PRKCB	HPPVL**TP**P-DQE
pelodiscus_sinensis	PRKCB	HPPVL**TP**P-DQE
xenopus_tropicalis	prkcb	HPPVL**TP**P-DHE
drosophila_melanogaster	Pkc53E	EKTDL**TP**T-DKV
drosophila_melanogaster	Pkcdelta	ERVRL**TP**I-DKE
drosophila_melanogaster	inaC	EKTDL**TP**T-DKL
caenorhabditis_elegans	pkc-2	LPTKM**TP**P-DWE
caenorhabditis_elegans	tpa-1	EKAAL**TP**VHDKN

ENSRNOP00000003697[Prpsap2]@227

rattus_norvegicus	**Prpsap2**	VDGRH**SP**PMV---R
gallus_gallus	PRPSAP2	VDGRH**SP**PTA---K
pelodiscus_sinensis	PRPSAP2	VDGRH**SP**PTV---K
anolis_carolinensis	PRPSAP2	VDGRH**SP**PTA---K
danio_rerio	prpsap2	VDGRH**SP**PTV---K
petromyzon_marinus	prpsap2	DDGRH**SP**PPIVMKN
drosophila_melanogaster	CG2246	VDGRY**SP**PPT---S
drosophila_melanogaster	CG6767	--------------
caenorhabditis_elegans	W04G3.5	EDGRQ**SP**PPN---V
caenorhabditis_elegans	R151.2	--------------

ENSRNOP00000020720[Slc32a1]@97

rattus_norvegicus	**Slc32a1**	LPPS---------G**S**KDQAV
gallus_gallus	SLC32A1	LPPS---------A**S**KDA--
pelodiscus_sinensis	SLC32A1	LPRS---------A**T**KDE--
anolis_carolinensis	SLC32A1	LPPS---------G**S**KEQ--
xenopus_tropicalis	slc32a1	-PSS---------A**S**KDE--
danio_rerio	slc32a1	-PPS---------A**S**KDG--
petromyzon_marinus	slc32a1	RNSGG--------GGGDG--
drosophila_melanogaster	VGAT	QGSI---------A**S**EGS--
caenorhabditis_elegans	unc-47	ESVVSEQPQKDDINKQE--
