# Supplementary material for: Evolutionary analysis of proline-directed phosphorylation sites in the mammalian growth cone identified using phosphoproteomics
Source: Mol Brain. 2019 May 31;12:53. doi: 10.1186/s13041-019-0476-x (PMC6545026; doi:10.1186/s13041-019-0476-x)
Supplement: Supplementary file 2 — Methods and the legend to Figure S1. (DOCX 16 kb) [file 13041_2019_476_MOESM2_ESM.docx]

**Supplemental information**

**Methods**

**Evolutionary analysis of phosphorylated sites using bioinformatics.** With a custom JavaScript program, all data for the phosphopeptides were processed on the server, and then the resulting data were sorted and summarized according to the kinases. The frequency of the kinases was calculated by the counts of kinases multiplied by the counts of peptides. The frequency was further divided into P- and non-P-directed sites. To evaluate evolutionary conservation of the phosphorylation sites, we extracted the orthologous genes of the following 10 selected organisms from the comparative genome analysis data in Ensembl version 75: *C. elegans* and *Drosophila melanogaster* as non-vertebrates; and *Petromyzon marinus*, *Danio rerio*, *Xenopus tropicalis*, *Anolis carolinensis*, *Pelodiscus sinensis*, *Gallus gallus*, *Rattus norvegicus,* *and Homo sapiens (human)* as vertebrates [13]. From the multiple alignment data of these organisms performed by Ensembl, we counted the conserved phosphosite residues of the rat phosphoproteomics data in vertebrates and non-vertebrates. In addition, we predicted the protein kinases that phosphorylate the phosphorylation sites with the KinasePhos 2.0 server [14]. From the prediction results, the top hit kinases with a score ≥0.5 were extracted. The number of phosphorylation sites conserved in vertebrates, but not in invertebrates, was divided by the total number of these sites that were conserved in vertebrates and non-vertebrates. When the score threshold for the rat phosphoproteomics data is given, conserved phosphorylation sites possessing a score more than the threshold showed a similar pattern. In addition, when we counted the number of genes containing phosphorylation sites instead of the genes themselves, the tendency was the same.

**Figure S1. Alignment of the P-directed GCM phosphoproteins emerging from invertebrates.** SP/TP residues are shown in *red*. The alignment of the phosphorylated sites in *Rattus norvegicus* (rat), *Gallus gallus* (chicken), *Pelodiscus sinensis* (turtle), *Anolis carolinensis* (green anole), *Xenopus tropicalis* (frog), *Danio rerio* (zebrafish), *Drosophila melanogaster* (fruit fly), and *Caenorhabditis elegans* (nematode). The data of vertebrates and invertebrates are separated by the horizontal line. If the corresponding gene was not found in *C. elegans*, the *C. elegans* data were not listed.
